# Identification of Genes Involved in Chemoreception in *Plutella xyllostella* by Antennal Transcriptome Analysis

**DOI:** 10.1038/s41598-017-11646-7

**Published:** 2017-09-20

**Authors:** Shiyong Yang, Depan Cao, Guirong Wang, Yang Liu

**Affiliations:** 1 Anhui Provincial Key Laboratory of the Conservation and Exploitation of Biological Resources, College of Life Sciences, Anhui Normal University, Wuhu, 241000 China; 2 State Key Laboratory for Biology of Plant Diseases and Insect Pests, Institute of Plant Protection, Chinese Academy of Agricultural Sciences, Beijing, 100193 China

## Abstract

Perception of environmental and habitat cues is of significance for insect survival and reproduction. Odor detection in insects is mediated by a number of proteins in antennae such as odorant receptors (ORs), ionotropic receptors (IRs), odorant binding proteins (OBPs), chemosensory proteins (CSPs), sensory neuron membrane proteins (SNMPs) and odorant degrading enzymes. In this study, we sequenced and assembled the adult male and female antennal transcriptomes of a destructive agricultural pest, the diamondback moth *Plutella xyllostella*. In these transcriptomes, we identified transcripts belonging to 6 chemoreception gene families related to ordor detection, including 54 ORs, 16 IRs, 7 gustatory receptors (GRs), 15 CSPs, 24 OBPs and 2 SNMPs. Semi-quantitative reverse transcription PCR analysis of expression patterns indicated that some of these ORs and IRs have clear sex-biased and tissue-specific expression patterns. Our results lay the foundation for future characterization of the functions of these *P*. *xyllostella* chemosensory receptors at the molecular level and development of novel semiochemicals for integrated control of this agricultural pest.

## Introduction

Olfaction plays a pivotal role in intra- and inter-specific interactions by directing insects towards food or prey, mating partners, oviposition sites, and away predators as well as toxic compounds^1^. The specialized organ for olfaction in insects is the antenna, on which hair-like, multi-pore sensilla are situated and peripheral olfactory signaling events occur. Olfactory receptor neurons (ORNs) and their auxiliary structures are located at the roots of the antennae^2^, and the entire olfactory system is dependent to a great extent on receptors expressed at the peripheral ORNs. Starting with perception of semiochemicals and ultimately ending with the translation of olfactory signals into behavior, the entire process requires orchestration of the insect’s sophisticated olfactory system at various levels. Several types of olfactory proteins are believed to participate in the selective detection and, once they have conveyed information, the rapid inactivation of trace amount of odorants, i.e. odorant receptors (ORs), ionotropic receptors (IRs), gustatory receptors (GRs), odorant binding proteins (OBPs), chemosensory proteins (CSPs) and sensory neuron membrane proteins (SNMPs)^3^.

Insect ORs are seven-transmembrane domain proteins with a reversed topology compared to the G-protein coupled ORs in vertebrates^4,5^. ORs play a central role in converting semiochemicals into electrical signal, functioning as a heterodimer with a divergent, conventional ORx and a highly conserved noncanonical OR co-receptor Orco in fruit fly, OR2 in moths and OR7 in mosquitoes^3^. The *OR* genes are expressed in the olfactory neurons housed within the olfactory sensilla (found mainly on the antenna)^6^.

GRs are also seven-transmembrane domain proteins, but they are more ancient than ORs. GR genes are expressed in the gustatory neurons housed within the gustatory sensilla (found on the labia, maxillary palps, antennae, legs and genitalia)^7^. GRs can respond to tastants such as sugars, bitter substances, CO_2_ and some contact pheromones^8–11^.

IRs belong to the ionotropic glutamate receptor (iGluR)-like protein family and can be activated by small molecules like acetates and amine-like volatile compounds^12,13^. It has been proven that IRs are involved in chemosensation^14,15^ and other functions, i.e. regulation of the circadian clock in *Drosophila melanogaster* ^16^ and induction of physical defense in *Daphnia pulex* ^17^. IRs usually contain three transmembrane domains (TMDs), a bipartite ligand-binding domain with two lobes and one ion channel, and have been proposed to act as dimmers or trimers of subunits coexpressed in the same neuron^12^. However, they aren’t expressed in chemosensory neurons that express ORs or Orco^14^.

OBPs are the liaisons between external cues and ORs^18^, and they selectively bind hydrophobic odorant chemicals and transport them to the surface of the dendrites of ORNs^19–21^. OBPs also function in the recognition of specific odors through activation of the ORx/Orco complex^20^. Another class of odorant binding proteins, CSPs, are small soluble proteins expressed predominantly in the sensilum lymph as well as in non-olfactory tissues. It is clear that CSPs bind odorant or pheromone compounds^22–24^, but their olfactory mechanisms areas yet poorly studied.

SNMPs are insect membrane proteins that are known to associate with pheromone sensitive ORNs in Lepidoptera and Diptera^25^. There are two types of SNMPs, SNMP1 and SNMP2^25^. In moth, the subtype SNMP1 is coexpressed with pheromone receptors (PRs) in pheromone-responsive neurons^25^, whereas the subtype SNMP2 is confined to sensilla support cells^25–28^.

The diamondback moth, *Plutella xylostella* (Lepidoptera: Plutellidae), is a destructive insect pest distributed worldwide that can cause considerable damage in cruciferous crops. It is estimated that the total loss caused by *P*. *xylostella* is about US$4-5 billion annually^29^. Although a bioinformatics analysis of the whole-genome sequence has explained the evolutionary success of *P*. *xylostella* with regard to its expansion in gene families associated with the perception and detoxification of plant defense compounds/insecticides at the genetic and molecular levels^30^, the peripheral olfactory mechanisms that contribute to the fitness of this insect pest remain poorly understood. Identification of genes expressed in the antennae will supply baseline information to understand their likely function in odorant perception in *P*. *xylostella* and insects adaptation to various host plants.

In the present study, we sequenced and analyzed the antennal transcriptome of *P*. *xylostella* adults using second-generation high-throughput Illumina RNA sequencing (RNA-seq). The purpose of our study was to identify olfaction-related genes which might be targets as a part of pest control strategies of this insect pest species that devastates cruciferous vegetables. We identified 118 candidate chemosensory genes encoding 54 ORs, 16 IRs, 7 GRs, 15 CSPs, 24 OBPs and 2 SNMPs. The sex-biased and tissue-specific expression patterns of 54 ORs and 16 IRs was also determined by semi-quantitative reverse transcription PCR. We reported the protein sequences of these chemosensory genes in Supplementary Dataset File.

## Results

### Sequencing and unigene assembly

By using Hiseq. 2000 sequencing approach, a total of 60,041,232 and 59,753,272 raw reads were obtained from the *P*. *xylostella* female and male antennae samples, respectively. After removing low quality and adaptor reads, female and male antennae yielded 54,430,716 and 54,059,300 clean reads and 4,898,764,440 nt and 4,865,337,000 nt clean nucleotides, respectively. After initial assembly, 124,488(mean length 278 nt) and 132,190 contigs (mean length 268 nt) were obtained from the female and male antennae libraries, respectively. Next, 62,278 female (mean length 555 nt) and 63,928 male unigenes (mean length 531 nt) were generated after contig connecting. These two unigene sets were then pooled together for further clustering, which yielded a final set of 59,844unigenesconsisting of 18,570 distinct clusters and 41,274 distinct singletons. The mean length of these unigenes was 660 nt, and N50 was 979 nt (Table 1).

**Table 1:** Summary of the *Pluttela xylostella* transcriptome assembly.

	Sample	Total Number	Total Length (nt)	Mean Length (nt)	N50 (nt)	Consensus Sequences	Distinct Clusters	Distinct Singletons
**Contig**	Female	124,488	34,667,373	278	403	—	—	—
	Male	132,190	35,402,665	268	369	—	—	—
**Unigene**	Female	62,278	34,543,989	555	829	62,278	16,328	45,950
	Male	63,928	33,941,348	531	761	63,928	15,969	47,959
	All	59,844	39,492,885	660	979	59,844	18,570	41,274

### Identification of candidate chemosensory receptors: ORs and GRs

**Figure 1:**
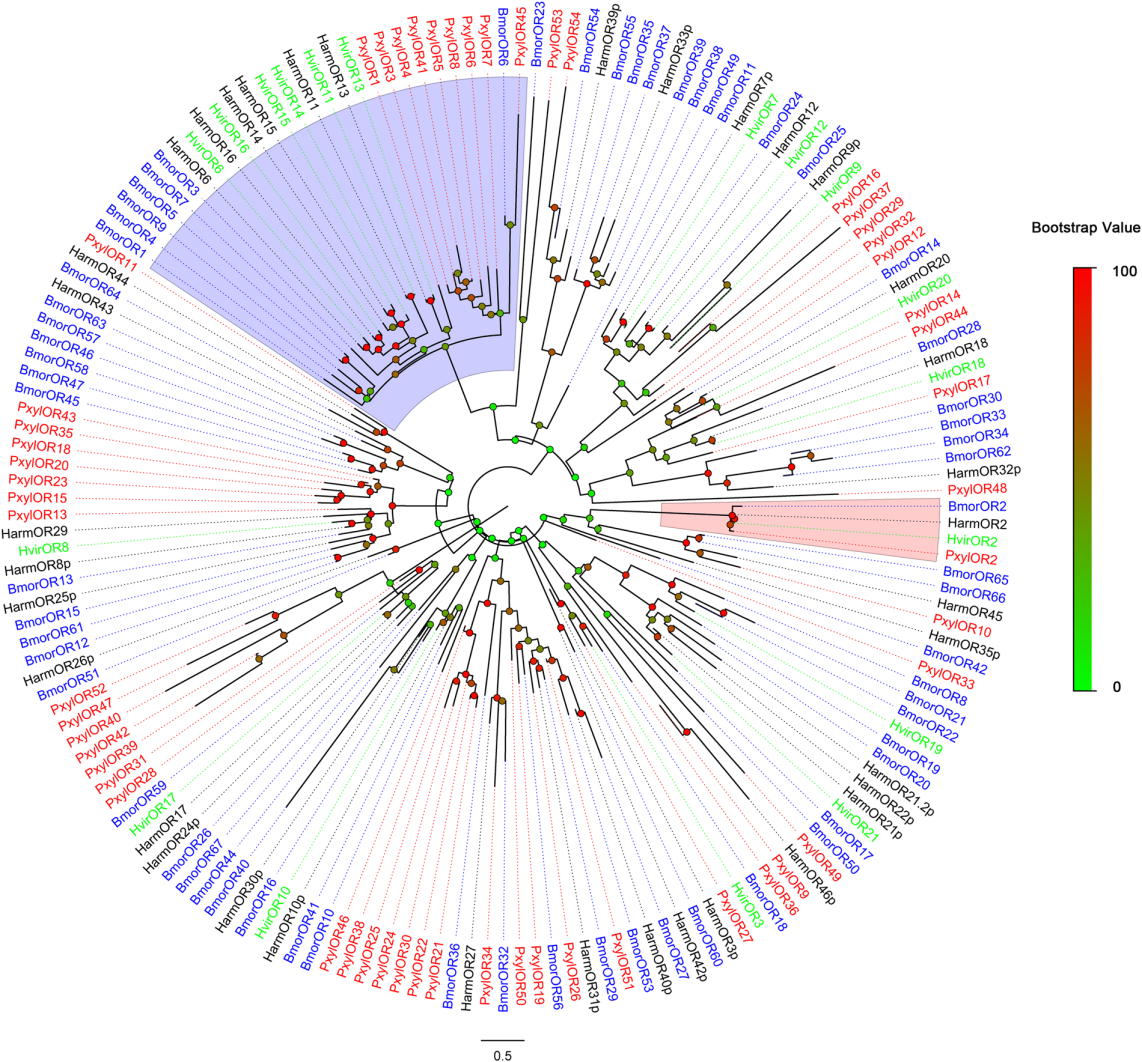
Phylogenetic tree of candidate Lepidopteran ORs, including the PR and Orco clades. Pxyl: *P*. *xylostella* (red), Harm: *Helicoverpa armigera* (black), Hvir: *Heliothis virescens* (green), Bmor: *Bombyx mori* (blue). The clade shaded in blue indicates the Orco clade. The clade shaded in red indicates the PBP clade. The bootstrap value for phylogenetic tree construction is 1000.

All the unigenes were searched by blastx against nr database and further by tblastn using 63 ORs from *B*. *mori* as queries, 54 candidate OR genes were identified (Table 2). Of these, 23 were predicted to have full-length open reading frames (ORFs). The length of these 23 OR genes ranges from 376 to 473 amino acid residues, and the encoded proteins are estimated to have 5–7 TMDs, which is characteristic of typical insect ORs. The remaining 31 OR genes code for at least 163 amino acids and are predicted to have more than one TMD. A phylogenetic analysis was then performed using our candidate ORs and the ORs from other Lepidopteran insects including *H*. *armigera*, *H*. *virescens* and *B*. *mori* (Fig. 1).

**Table 2:** Candidate olfactory receptor and gustatory receptor unigenes.

Unigene reference	Name	Length(bp)	ORF(aa)	Blastx best hit (Reference/Name/Species)	E value	Identity	TMD (No)	Status
**Co-receptor**								
Unigene25399	PxylOR2	2187	473	dbj|BAG71421.2| olfactory receptor-2 [*Plutella xylostella*]	0	1	7	Complete
**Pheromone receptors**								
CL4851.Contig2	PxylOR1	1800	422	dbj|BAG71420.1| olfactory receptor-1 [*P*. *xylostella*]	0	1	6	Complete
CL902.Contig17	PxylOR3	1650	402	dbj|BAG71425.2| olfactory receptor [*P*. *xylostella*]	0	0.99	5	Complete
CL902.Contig2	PxylOR4	1595	402	dbj|BAG71426.1| olfactory receptor [*P*. *xylostella*]	0	0.95	7	Complete
CL902.Contig3	PxylOR5	1630	404	dbj|BAG71426.1| olfactory receptor [*P*. *xylostella*]	0	0.82	6	Complete
Unigene18038	PxylOR6	1584	424	dbj|BAG71426.1| olfactory receptor [*P*. *xylostella*]	3.00E-129	0.48	7	Complete
CL3732.Contig1	PxylOR7	1415	424	dbj|BAG71425.2| olfactory receptor [*P*. *xylostella*]	5.00E-107	0.42	7	Complete
CL3275.Contig3	PxylOR8	1717	427	dbj|BAG71425.2| olfactory receptor [*P*. *xylostella*]	3.00E-129	0.63	6	Complete
CL902.Contig18	PxylOR41	580	193	dbj|BAG71426.1|olfactory receptor [*P*.*xylostella*]	1.00E-83	0.77	1	5′, 3′ lost
Unigene8020	PxylOR45	568	189	ref|NP_001036928.1| olfactory receptor 6 [*Bombyx mori*]	3.00E-27	0.33	3	5′, 3′ lost
**Olfactory receptors**								
CL1915.Contig1	PxylOR9	1466	449	ref|NP_001116817.1| olfactory receptor-like [*B*. *mori*]	5.00E-145	0.59	6	Complete
CL1947.Contig5	PxylOR10	1602	428	gb|AFC91732.1| putative odorant receptor OR24 [*Cydia pomonella*]	4.00E-127	0.45	7	Complete
Unigene8291	PxylOR11	1369	421	ref|NP_001166621.1| olfactory receptor 64 [*B*. *mori*]	2.00E-73	0.5	6	Complete
Unigene25275	PxylOR12	1340	420	gb|AFC91725.1| putative odorant receptor OR17 [*C*. *pomonella*]	1.00E-97	0.51	6	5′ lost
CL6791.Contig2	PxylOR13	1396	415	emb|CAD31949.1| putative chemosensory receptor 8 [*Heliothis virescens*]	1.00E-124	0.49	7	5′ lost
CL6176.Contig1	PxylOR14	1451	412	emb|CAG38121.2| putative chemosensory receptor 20 [*H*. *virescens*]	1.00E-137	0.53	7	Complete
CL3142.Contig2	PxylOR15	1579	409	ref|NP_001091789.1| olfactory receptor 15 [*B*. *mori*]	4.00E-76	0.39	7	5′ lost
CL2401.Contig2	PxylOR16	1257	405	gb|AFC91721.1| putative odorant receptor OR12 [*C*. *pomonella*]	2.00E-166	0.58	6	Complete
Unigene19920	PxylOR17	1722	399	gb|AFC91726.1| putative odorant receptor OR18 [*C*. *pomonella*]	1.00E-120	0.45	7	Complete
Unigene3520	PxylOR18	1367	396	tpg|DAA05974.1| TPA_exp: odorant receptor 15 [*B*. *mori*]	3.00E-94	0.4	7	Complete
Unigene5731	PxylOR19	1294	395	ref|NP_001166617.1| olfactory receptor 56 [*B*. *mori*]	8.00E-145	0.53	7	Complete
CL6714.Contig1	PxylOR20	1362	393	ref|NP_001091789.1| olfactory receptor 15 [*B*. *mori*]	1.00E-80	0.37	6	Complete
CL2099.Contig4	PxylOR21	1751	393	ref|NP_001166892.1| olfactory receptor 36 [*B*. *mori*]	4.00E-34	0.24	7	Complete
CL2099.Contig5	PxylOR22	1606	393	ref|NP_001166892.1| olfactory receptor 36 [*B*. *mori*]	9.00E-39	0.26	7	Complete
CL2363.Contig1	PxylOR23	1265	392	tpg|DAA05974.1| TPA_exp: odorant receptor 15 [*B*. *mori*]	5.00E-90	0.4	7	Complete
CL918.Contig2	PxylOR24	1222	391	ref|NP_001166892.1| olfactory receptor 36 [*B*. *mori*]	5.00E-35	0.27	7	Complete
Unigene25128	PxylOR25	1219	389	ref|NP_001166892.1| olfactory receptor 36 [*B*. *mori*]	5.00E-47	0.3	6	Complete
Unigene5953	PxylOR26	1156	385	gb|EHJ78030.1| olfactory receptor 29 [*Danaus plexippus*]	6.00E-141	0.63	6	3′ lost
Unigene5680	PxylOR27	1314	376	gb|EHJ64733.1| olfactory receptor 18 [*D*. *plexippus*]	2.00E-136	0.55	7	Complete
CL1359.Contig2	PxylOR28	1737	359	ref|NP_001091790.1| candidate olfactory receptor [*B*. *mori*]	1.00E-71	0.33	6	5′ lost
CL6074.Contig2	PxylOR29	1214	356	emb|CAG38113.1| putative chemosensory receptor 12 [*H*. *virescens*]	9.00E-65	0.38	6	5′, 3′lost
CL2099.Contig6	PxylOR30	1140	301	ref|NP_001166892.1| olfactory receptor 36 [*B*. *mori*]	2.00E-34	0.28	5	5′ lost
Unigene14039	PxylOR31	949	279	ref|NP_001166611.1| olfactory receptor 59 [*B*. *mori*]	3.00E-56	0.38	2	5′ lost
Unigene11354	PxylOR32	835	277	gb|EHJ65925.1| olfactory receptor 12 [*D*. *plexippus*]	8.00E-62	0.45	4	5′, 3′lost
CL741.Contig1	PxylOR33	927	272	gb|AFC91717.1| putative odorant receptor OR7, partial [*C*. *pomonella*]	2.00E-41	0.4	4	5′ lost
Unigene600	PxylOR34	862	270	tpg|DAA05988.1| TPA_exp: odorant receptor 32 [*B. mori*]	2.00E-30	0.33	4	3′ lost
CL4545.Contig1	PxylOR35	824	269	tpg|DAA05974.1| TPA_exp: odorant receptor 15 [*B. mori*]	3.00E-56	0.39	5	5′ lost
Unigene17021	PxylOR36	768	252	gb|ACH69152.1| olfactory receptor 49 [*B*. *mori*]	8.00E-120	0.68	5	5′ lost
Unigene21064	PxylOR37	706	235	gb|AFC91721.1| putative odorant receptor OR12 [*C*. *pomonella*]	4.00E-32	0.39	4	5′, 3′ lost
CL7033.Contig1	PxylOR38	646	215	ref|NP_001166892.1| olfactory receptor 36 [*B*. *mori*]	1.00E-28	0.37	3	5′, 3′ lost
Unigene25541	PxylOR39	613	204	gb|AFC91719.1| putative odorant receptor OR10 [*C. pomonella*]	3.00E-69	0.55	3	5′, 3′ lost
Unigene3305	PxylOR40	601	200	gb|AFC91724.1| putative odorant receptor OR16 [*C*. *pomonella*]	6.00E-70	0.66	4	5′, 3′ lost
Unigene21899	PxylOR42	581	193	ref|NP_001104832.2| olfactory receptor 16 [*B*. *mori*]	5.00E-70	0.66	3	5′, 3′ lost
CL4065.Contig1	PxylOR43	578	192	tpg|DAA05974.1| TPA_exp: odorant receptor 15 [*B*. *mori*]	6.00E-24	0.36	2	5′, 3′ lost
Unigene7439	PxylOR44	570	190	gb|ACC63240.1| olfactory receptor 20, partial [*Helicoverpa armigera*]	8.00E-32	0.37	4	5′, 3′ lost
Unigene21835	PxylOR46	654	187	gb|EFA09245.1| odorant receptor 14 [*Tribolium castaneum*]	1.00E-08	0.23	2	5′ lost
Unigene9201	PxylOR47	545	181	gb|ACM18061.1| putative odorant receptor OR3 [*Manduca sexta*]	8.00E-21	0.36	3	5′, 3′ lost
CL764.Contig1	PxylOR48	544	180	ref|NP_001091791.1| candidate olfactory receptor [*B*. *mori*]	2.00E-12	0.27	3	5′, 3′ lost
CL3314.Contig3	PxylOR49	797	177	ref|NP_001166611.1| olfactory receptor 59 [*B*. *mori*]	1.00E-17	0.31	3	5′, 3′ lost
Unigene27391	PxylOR50	531	177	gb|EHJ78030.1| olfactory receptor 29 [*Danaus plexippus*]	2.00E-38	0.49	3	5′, 3′ lost
Unigene23191	PxylOR51	522	174	ref|NP_001166893.1| olfactory receptor 27 [*B*. *mori*]	4.00E-65	0.55	4	5′, 3′ lost
Unigene5685	PxylOR52	809	170	dbj|BAH66323.1| olfactory receptor [*B*. *mori*]	3.00E-34	0.55	2	5′ lost
Unigene28136	PxylOR53	491	164	gb|AEF32141.1| odorant receptor [*S*. *exigua*]	5.00E-26	0.51	3	5′, 3′ lost
Unigene11787	PxylOR54	490	163	ref|NP_001166616.1| olfactory receptor 54 [*B*. *mori*]	1.00E-30	0.47	1	5′, 3′ lost
**Gustatory receptors**								
Unigene22668	PxylGR1	1588	392	ref|XP_001848097.1| gustatory receptor 22 [*Culex quinquefasciatus*]	0	0.71	7	Complete
Unigene15579	PxylGR2	958	227	dbj|BAK52798.1| gustatory receptor 66 [*B*. *mori*]	9.00E-32	0.35	4	5′ lost
CL3914.Contig2	PxylGR3	507	168	gb|ABY40622.1| gustatory receptor [*T*. *castaneum*]	2.00E-50	0.62	2	5′, 3′ lost
Unigene32005	PxylGR4	343	114	ref|NP_001233217.1| gustatory receptor 68 [*B*. *mori*]	3.00E-14	0.38	1	5′, 3′ lost
Unigene6419	PxylGR5	328	109	emb|CAD31850.1| putative chemosensory receptor 1 [*H*. *virescens*]	2.00E-21	0.48	2	5′, 3′ lost
Unigene34245	PxylGR6	264	88	dbj|BAK52798.1| gustatory receptor 66 [*B*. *mori*]	3.00E-10	0.49	0	5′, 3′ lost
Unigene19491	PxylGR7	723	240	emb|CAD31850.1| putative chemosensory receptor 1 [*H*. *virescens*]	8.00E-31	0.35	3	5′, 3′ lost

The OR co-receptor gene was easily identified because of extremely high conservation among species compared to other chemosensory receptors. Similar to other insect ORs, most *P*. *xylostella* (Pxyl) ORs are highly divergent and share low similarity with other Lepidopteran insect ORs, including ORs from *H*. *armige*ra, *H*. *virescens* and *B*. *mori*. However, nine PxylORs had 33%~100% identity to previously characterized PRs from *P*. *xylostella* and *B*. *mori*. They formed a single subgroup in a phylogenetic tree of Lepidopteran ORs (Fig. 1). Seven of these nine PxylORs (PxylOR1 andPxylOR3-8) were predicted to have full-length ORFs. Two short sequences (PxylOR41and PxylOR45) were also clustered in the PR branch. PxylOR41 has high similarity to PxylOR4, and PxylOR45 has relatively high similarity to BmorOR6. 12 of the remaining PxylORs were clustered with their Lepidopteran orthologous genes in the phylogenetic tree. But most PxylORs appeared to be distantly related to the known insect ORs (Fig. 1). We named the Orco unigene PxylOR2 and the 7full-length candidate PR unigenes PxylOR1 and PxylOR3-PxylOR8. The other 46 OR unigenes were ranked in order of decreasing ORF length and named PxylOR9-PxylOR54. We also identified 7 candidate GRs and named them as PxylGR1-PxylGR7.

### Identification of candidate IRs

**Figure 2:**
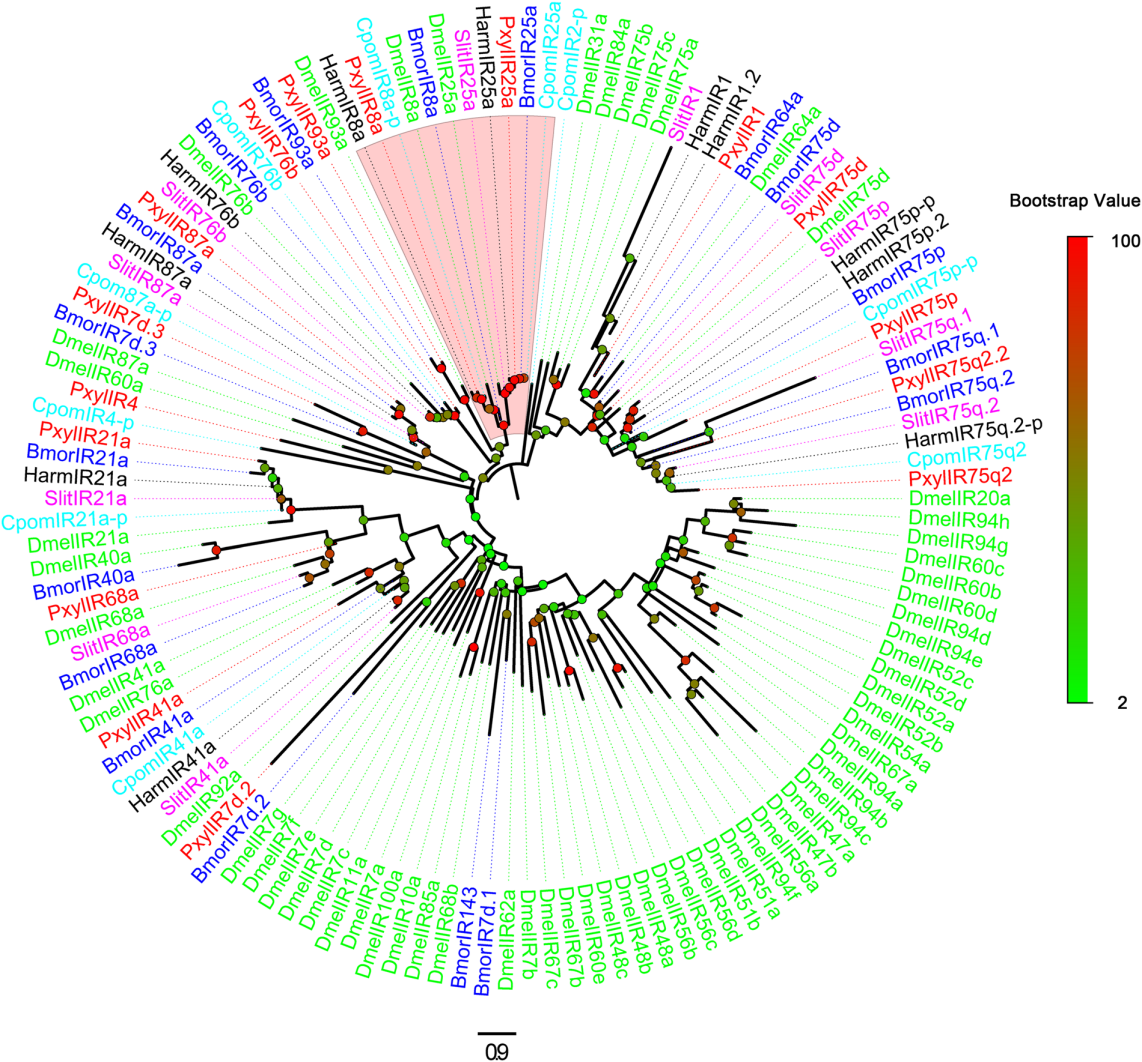
Phylogenetic tree of candidate IRs from *Pluttela xylostella* and other insects. Pxyl: *P*. *xylostella* (red), Harm: *Helicoverpa armigera* (black), Hvir: *Heliothis virescens* (green), Bmor: *Bombyx mori* (blue), Slit: *Spodoptera littoralis* (purple), Cpom: *Cydia pomonella* (cyan).The clade shaded in red indicates the IR8a/IR25a clade. The bootstrap value for phylogenetic tree construction is 1000.

IR sequences in the *P*. *xylostella* antennal transcriptome were identified based on similarity to known IRs of Lepidopteran insects, *B*. *mori*, *C*. *pomonella*, *H*. *armigera*, *H*. *virescens* and *S*. *littoralis*. Sixteen candidate IRs were identified by bioinformatic analysis, and five unigenes were predicted to have a full-length ORFs. The insect IRs typically have three TMDs. Of the 16 candidates IRs, 15 are predicted to have at least one TMD (Table 3). Twelve of the 16 putative IRs are at least 48% identical to the corresponding Lepidopteran orthologous IRs in *S*. *littoralis* and *C*. *pomonella*. The remaining four unigenes have relatively low similarity to other insect IRs: CL2177.Contig2 has 35% identity with IR1 of *S*. *littoralis*, unigene13888 has 31% identity with IR75 of *C*. *pomonella*, CL4692.Contig1 has only 25% identity with IR60a of *D*. *melanogaster*, and CL5979.Contig2 has only 24% identity with IR7c of *D*. *melanogaster* (Table 3). Phylogenetic analyses suggested that the prediction of IRs was credible. In a neighbor-joining tree of insect IRs, all candidate PxylIRs were clustered in a separate clade with their Lepidopteran orthologs (Fig. 2). All of these 16 candidate IR unigenes were named based on their homology to known IRs. For example, the IR Unigene 19385 has 55% similarity withIR75q2 and CL1791. Contig1 had 64% similarity to IR75q2. So, we named Unigene 19835 PxylIR75q2.2.

**Table 3:** Candidate ionotropic receptor unigenes.

Unigene reference	Name	Length (bp)	ORF(aa)	Blastx best hit (Reference/Name/Species)	E value	Identity	TMD (No)	Status
CL2177.Contig2	PxylIR1	1559	483	gb|ADR64688.1| putative chemosensory ionotropic receptor IR1 [*Spodoptera littoralis*]	5.00E-70	0.35	3	5′ lost
Unigene13888	PxylIR4	1133	345	gb|AFC91756.1| putative ionotropic receptor IR75, partial [*Cydia pomonella*]	6.00E-17	0.31	0	3′ lost
CL4692.Contig1	PxylIR7d.2	1717	504	ref|NP_611901.1| ionotropic receptor 60a [*Drosophila melanogaster*]	4.00E-31	0.25	3	3′ lost
CL5979.Contig2	PxylIR7d.3	1624	330	gb|AFC91764.1|ionotropic receptor 7c, isoform A [*D*. *melanogaster*]	1.00E-11	0.24	2	3′ lost
Unigene18533	PxylIR8a	3047	907	gb|AFC91764.1| putative ionotropic receptor IR8a, partial [*C*. *pomonella*]	0	0.79	4	Complete
CL721.Contig4	PxylIR21a	2576	858	gb|ADR64678.1| putative chemosensory ionotropic receptor IR21a [*S*. *littoralis*]	0	0.65	4	5′, 3′ lost
Unigene25424	PxylIR25a	3139	932	gb|AFC91757.1| putative ionotropic receptor IR25a [*C*. *pomonella*]	0	0.89	3	Complete
Unigene25124	PxylIR41a	994	330	gb|AFC91758.1| putative ionotropic receptor IR41a [*C*. *pomonella*]	3.00E-102	0.53	1	5′, 3′ lost
Unigene255	PxylIR68a	869	289	gb|ADR64682.1| putative chemosensory ionotropic receptor IR68a [*S*. *littoralis*]	4.00E-103	0.67	3	5′, 3′ lost
CL6386.Contig3	PxylIR75d	1884	593	gb|ADR64683.1| putative chemosensory ionotropic receptor IR75d [*S*. *littoralis*]	4.00E-138	0.48	3	Complete
Unigene8511	PxylIR75p	1356	287	gb|AFC91755.1| putative ionotropic receptor IR75p, partial [*C*. *pomonella*]	3.00E-127	0.79	3	5′ lost
CL1791.Contig1	PxylIR75q2	1441	410	gb|AFC91752.1| putative ionotropic receptor IR75q2 [*C*. *pomonella*]	1.00E-163	0.64	1	3′ lost
Unigene19385	PxylIR75q2.2	1806	591	gb|AFC91752.1| putative ionotropic receptor IR75q2 [*C*. *pomonella*]	0	0.55	3	5′ lost
CL3281.Contig2	PxylIR76b	1790	551	gb|AFC91765.1| putative ionotropic receptor IR76b [*C*. *pomonella*]	0	0.64	3	Complete
Unigene2044	PxylIR87a	1901	633	gb|AFC91760.1| putative ionotropic glutamate receptor 87a, partial [*C*. *pomonella*]	5.00E-167	0.73	4	5′, 3′ lost
Unigene5567	PxylIR93a	2763	878	gb|AFC91753.1| putative ionotropic receptor IR93a, partial [*C*. *pomonella*]	2.00E-174	0.74	3	Complete

### Identification of putative OBPs

**Figure 3:**
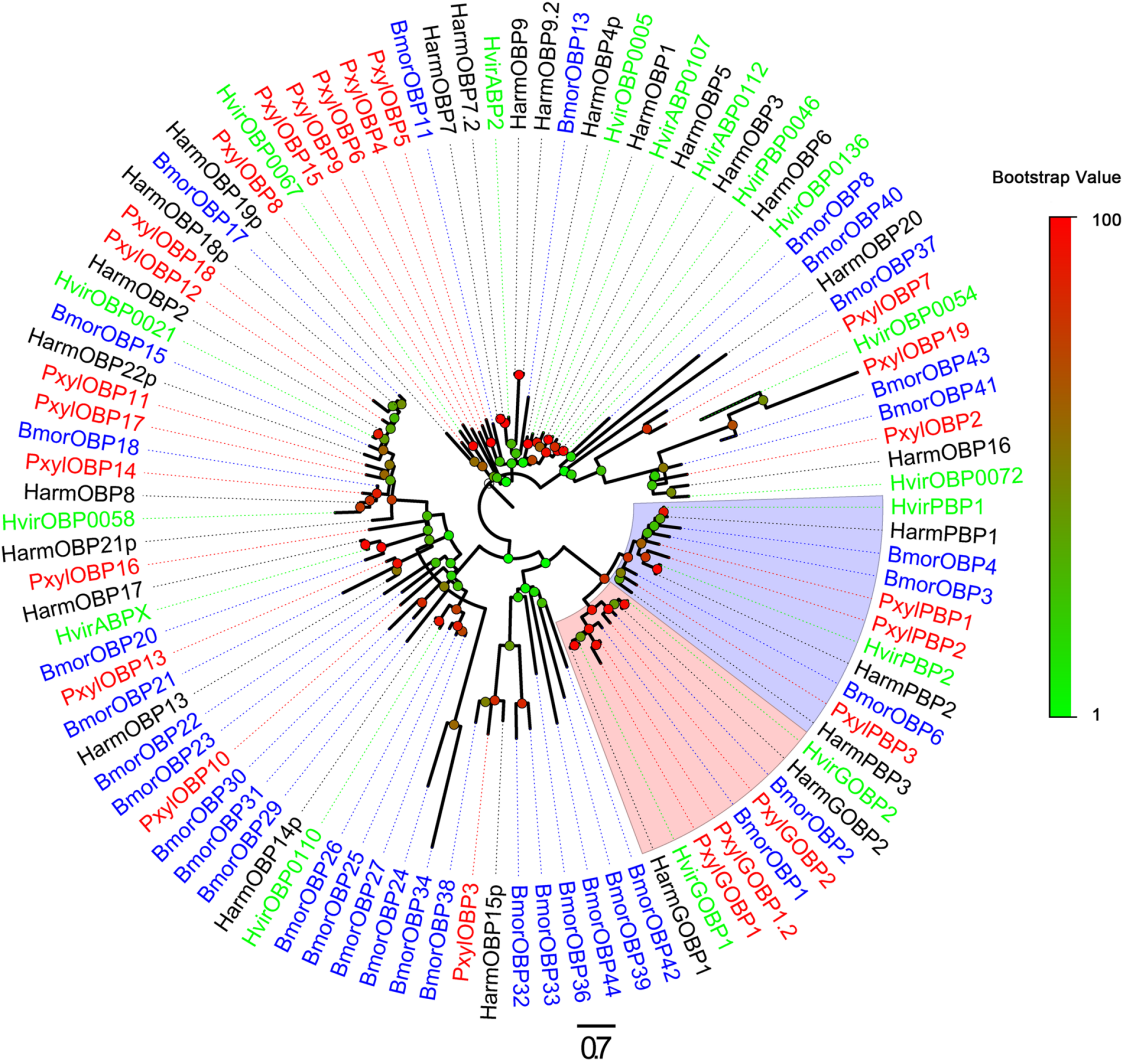
Phylogenetic tree of candidate Lepidopteran OBPs, including the GOBP and PBP clades. Pxyl: *Pluttela xylostella* (red), Harm: *Helicoverpa armigera* (black), Hvir: *Heliothis virescens* (green), Bmor: *Bombyx mori* (blue). The clade shaded in blue indicates the PBP clade. The clade shaded in red indicates the GOBP clade. The bootstrap value for phylogenetic tree construction is 1000.

We identified 24 unigenes encoding OBPs from the antennal transcriptome of *P*. *xylostella*, including 3pheromone binding proteins (PBPs) and 3 general odorant binding proteins (GOBPs) (Table 4). Twenty-two of these 24 unigenes were predicted to have signal peptides, and 19 have full length ORFs. Signal peptide sequences were not detected in the remaining two putative OBPs due to incomplete N-terminal sequences. All 24 putative OBPs had high similarity to known Lepidopteran OBPs. The PBP and GOBP sequences were clustered in a separate clade in the OBP neighbor-joining tree (Fig. 3). Three candidate OBPs were classified into a PBP subgroup in the phylogenetic tree. They share 66%~100% similarity with previously characterized Lepidopteran PBPs and thus were named PBPs. We also found two GOBPs in the antennal transcriptome of *P*. *xylostella* and named them PxylGOBP1 and GOBP2. A new GOBP (PxylGOBP1.2) was identified that has 77% identity with PxylGOBP1. It was clustered in the GOBP clade and distinguished from other OBPs in the phylogenetic tree. The other 18 candidate OBPs are obviously distinct from the PBP and GOBP clades and have relatively lower similarity to OBPs from other Lepidopteran insects. Most candidate OBP sequences, such as PxylOBP2, PxylOBP3, and PxylOBP7, are closely clustered with at least one Lepidopteran ortholog, in congruence with the blastx results. Some candidate OBP sequences such as PxylOBP6, PxylOBP9, PxylOBP11 and PxylOBP17 are not clustered with OBPs from other Lepidopteran insects (Fig. 3). A possible reason may be that the orthologs of these PxylOBPs have not been identified in other Lepidopteran insects.

**Table 4:** Candidate odorant binding protein unigenes.

Unigene reference	Gene name	Length (bp)	ORF (aa)	Blastx best hit (Reference/Name/Species)	E value	Identity	Signal peptide	Status
**Pheromone binding protein**								
Unigene8499	PxylPBP1	761	164	dbj|BAG71422.1| pheromone binding protein [*Plutella xylostella*]	5.00E-92	0.99	Yes	Complete
Unigene2096	PxylPBP2	845	172	gb|AAF06143.1|AF177661_1 pheromone binding protein [*Yponomeuta cagnagellus*]	3.00E-63	0.66	Yes	Complete
CL3437.Contig1	PxylPBP3	1322	164	gb|ACI28451.1| pheromone binding protein 1 [*P*. *xylostella*]	3.00E-88	0.95	Yes	Complete
**General odorant binding protein**								
CL5166.Contig1	PxylGOBP1	862	168	gb|ABW05104.1| general odorant-binding protein 1 [*P*. *xylostella*]	4.00E-97	0.93	Yes	Complete
CL3061.Contig1	PxylGOBP1.2	1003	166	gb|ABY71034.1| general odorant binding protein 1 [*P*. *xylostella*]	1.00E-70	0.77	Yes	Complete
CL3886.Contig3	PxylGOBP2	4230	163	gb|ABY71035.2| general odorant binding protein 2 [*P*. *xylostella*]	1.00E-90	1.00	Yes	Complete
**Other odorant binding protein**								
CL6467.Contig2	PxylOBP2	811	190	gb|EHJ77172.1| odorant binding protein [*Danaus plexippus*]	1.00E-40	0.41	Yes	Complete
Unigene10356	PxylOBP3	867	173	gb|ACF48467.1| pheromone binding protein female 1 [*Loxostege sticticalis*]	2.00E-37	0.66	Yes	Complete
Unigene103	PxylOBP4	1894	161	gb|AFD34177.1| odorant binding protein 1 *[Argyresthia conjugella*]	4.00E-30	0.48	Yes	Complete
Unigene6155	PxylOBP5	962	158	gb|AFD34177.1| odorant binding protein 1 [*A*.*conjugella*]	1.00E-22	0.42	Yes	Complete
CL1521.Contig2	PxylOBP6	2242	153	gb|ADK47525.1| odorant binding protein [*Manduca sexta*]	8.00E-23	0.40	Yes	Complete
Unigene25127	PxylOBP7	486	152	emb|CAS90127.1| odorant binding protein 3 precursor [*Bombyx mori*]	5.00E-44	0.58	Yes	3′ lost
CL5131.Contig2	PxylOBP8	531	149	gb|AER27561.1| odorant binding protein [*P*. *xylostella*]	3.00E-38	0.99	Yes	Complete
CL4848.Contig1	PxylOBP9	570	148	gb|EHJ67764.1| odorant-binding protein 5 [*D*. *plexippus*]	4.00E-15	0.37	Yes	Complete
CL2704.Contig3	PxylOBP10	736	143	gb|ACX53795.1| odorant binding protein [*Heliothis virescens*]	1.00E-14	0.33	Yes	Complete
Unigene10167	PxylOBP11	582	143	gb|AFD34180.1| odorant binding protein 3 [*A*. *conjugella*]	1.00E-42	0.60	Yes	Complete
CL4175.Contig1	PxylOBP12	1753	142	gb|EHJ65653.1| odorant-binding protein 1 [*D*. *plexippus*]	6.00E-51	0.77	Yes	Complete
Unigene26843	PxylOBP13	1086	141	gb|AFD34173.1| odorant binding protein 5 [*A*. *conjugella*]	6.00E-64	0.77	Yes	Complete
CL4228.Contig1	PxylOBP14	726	140	gb|AFD34175.1| odorant binding protein 4 [*A*. *conjugella*]	3.00E-55	0.72	Yes	Complete
Unigene21533	PxylOBP15	422	140	gb|ACX53756.1| odorant binding protein [*H*. *virescens*]	1.00E-37	0.52	Yes	5′, 3′ lost
Unigene15836	PxylOBP16	742	139	gb|AFD34182.1| odorant binding protein 6 [*A*. *conjugella]*	2.00E-47	0.66	Yes	Complete
CL2382.Contig4	PxylOBP17	444	129	gb|AFD34180.1| odorant binding protein 3 [*A*. *conjugella*]	9.00E-29	0.50	No	5′ lost
CL4528.Contig1	PxylOBP18	502	97	gb|AFG72998.1| odorant-binding protein 1 [*Cnaphalocrocis medinalis*]	2.00E-41	0.76	No	5′ lost
Unigene37282	PxylOBP19	228	64	gb|ACX53743.1| odorant binding protein [*H*. *virescens*]	2.00E-13	0.60	Yes	3′ lost

### Identification of candidate CSPs

**Figure 4:**
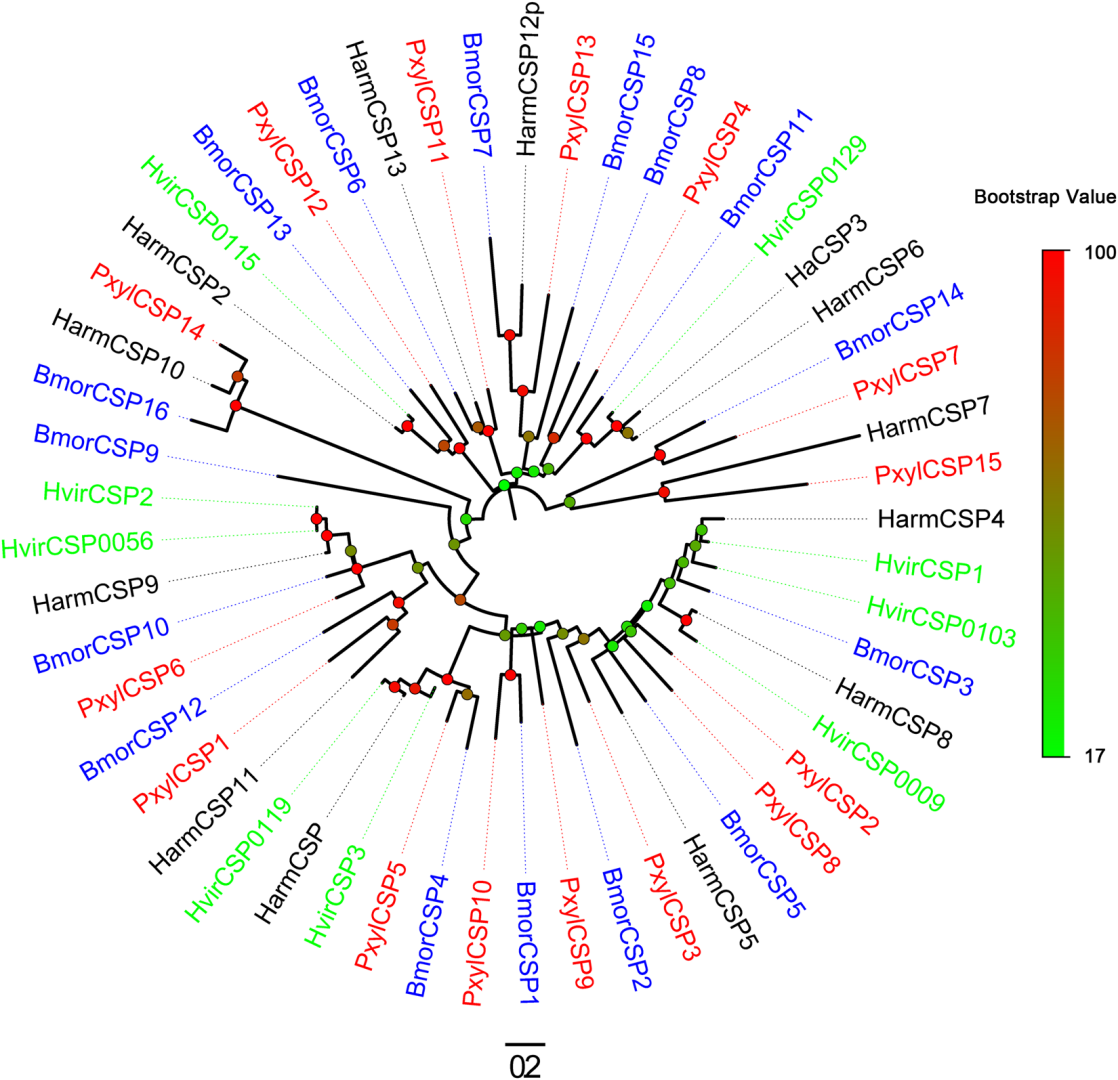
Phylogenetic tree of candidate Lepidopteran CSPs. Pxyl: *Pluttela xylostella* (red), Harm: *Heliocoverpa armigera* (black), Hvir: *Hethiothis virescens* (green), Bmor: *Bombyx mori* (blue). The bootstrap value for phylogenetic tree construction is 1000.

Bioinformatic analysis led to the identification of 15 different sequences encoding candidate CSPs (Table 5). All 15 unigenes were predicted to have signal peptides and 14 have a full length ORFs. Four candidate PxylCSPs (PxylCSP1-4) match the previously identified *P*. *xylostella* CSP sequences^31^. The other 11 candidate CSP sequences have at least 35% identity with known CSPs from other insects, and we named them according to the length of the coding region in descending order. In a neighbor-joining tree, all 15 sequences form a cluster with Lepidopteran orthologous genes (Fig. 4).

**Table 5:** Candidate chemosensory protein unigenes.

Unigene reference	Gene name	Length (bp)	ORF (aa)	Blastx best hit (Reference/Name/Species)	E value	Identity	Signal peptide	Status
Unigene7305	PxylCSP1	732	152	gb|ABM67686.1| chemosensory protein CSP1 [*Plutella xylostella*]	3.00E-84	0.99	Yes	Complete
Unigene12972	PxylCSP2	676	128	gb|ABM67687.1| chemosensory protein CSP2 [*P*. *ylostella*]	2.00E-71	0.98	Yes	Complete
Unigene5262	PxylCSP3	425	122	gb|ABM92663.1| chemosensory protein CSP3 [*P*. *ylostella*]	1.00E-65	0.99	Yes	3′ lost
CL1074.Contig1	PxylCSP4	1010	126	gb|ABM92664.1| chemosensory protein CSP4 [*P*. *ylostella*]	1.00E-66	1.00	Yes	Complete
Unigene1800	PxylCSP5	638	130	gb|AAK53762.1|AF368375_1 chemosensory protein [*Helicoverpa armigera*]	4.00E-53	0.73	Yes	Complete
CL574.Contig2	PxylCSP6	1632	130	dbj|BAF91712.1| chemosensory protein [*Papilio xuthus*]	8.00E-60	0.87	Yes	Complete
Unigene24730	PxylCSP7	552	127	dbj|BAG71921.1| chemosensory protein 13 [*P*. *xuthus*]	2.00E-43	0.69	Yes	Complete
Unigene10872	PxylCSP8	520	127	gb|ABM67689.1| chemosensory protein CSP2 [*Spodoptera exigua*]	9.00E-43	0.63	Yes	Complete
Unigene7440	PxylCSP9	657	123	gb|ACX53825.1| chemosensory protein [*Heliothis virescens*]	5.00E-43	0.64	Yes	Complete
Unigene7557	PxylCSP10	1041	123	dbj|BAF91711.1| chemosensory protein [*P*. *xuthus*]	4.00E-44	0.70	Yes	Complete
CL3090.Contig2	PxylCSP11	740	122	gb|EHJ73330.1| chemosensory protein [*Danaus plexippus*]	2.00E-51	0.77	Yes	Complete
Unigene21123	PxylCSP12	667	122	gb|AEX07265.1| CSP2 [*H*. *armigera*]	4.00E-42	0.56	Yes	Complete
CL1877.Contig3	PxylCSP13	886	120	gb|EHJ73328.1| chemosensory protein 11b [*D*. *plexippus*]	3.00E-27	0.49	Yes	Complete
Unigene21118	PxylCSP14	548	111	dbj|BAF91720.1| chemosensory protein [*P*. *xuthus*]	6.00E-45	0.81	Yes	Complete
CL2890.Contig2	PxylCSP15	672	110	ref|XP_001844687.1| chemosensory protein 1 [*Culex quinquefasciatus*]	1.00E-09	0.35	Yes	Complete

### Identification of candidate SNMPs

SNMPs were first identified in pheromone-sensitive neurons of Lepidoptera^31^ and are thought to function in pheromone detection^32^. Two kinds of SNMPs (SNMP1 and SNMP2) have been identified in insects and transcripts corresponding to both were found in the *P*. *xylostella* transcriptome. The sequence of CL2414Contig2 is identical to the PxylSNMP1sequence published in Genbank. CL242.Contig4 has 70% identity with SNMP2 of *O*. *furnacalis*, and we annotated this sequence as *P*. *xylostella* SNMP2 (Table 6).

**Table 6:** Candidate sensory neuron membrane protein unigenes.

Unigene reference	Gene name	Length (bp)	ORF (aa)	BLASTx best hit (Reference/Name/Species)	E value	Identity	Status
CL2414.Contig2	PxylSNMP1	2408	522	gi|301153754|gb|ADK66278.1| sensory neuron membrane protein 1 [*Plutella xylostella*]	0	1.00	Complete
CL242.Contig4	PxylSNMP2	2196	523	gi|312306074|gb|ADQ73891.1| sensory neuron membrane protein 2 [*Ostrinia furnacalis*]	0	0.70	Complete

### Tissue- and sex-specific expression of candidate OR and IR genes in *P*. *xylostella*

**Figure 5:**
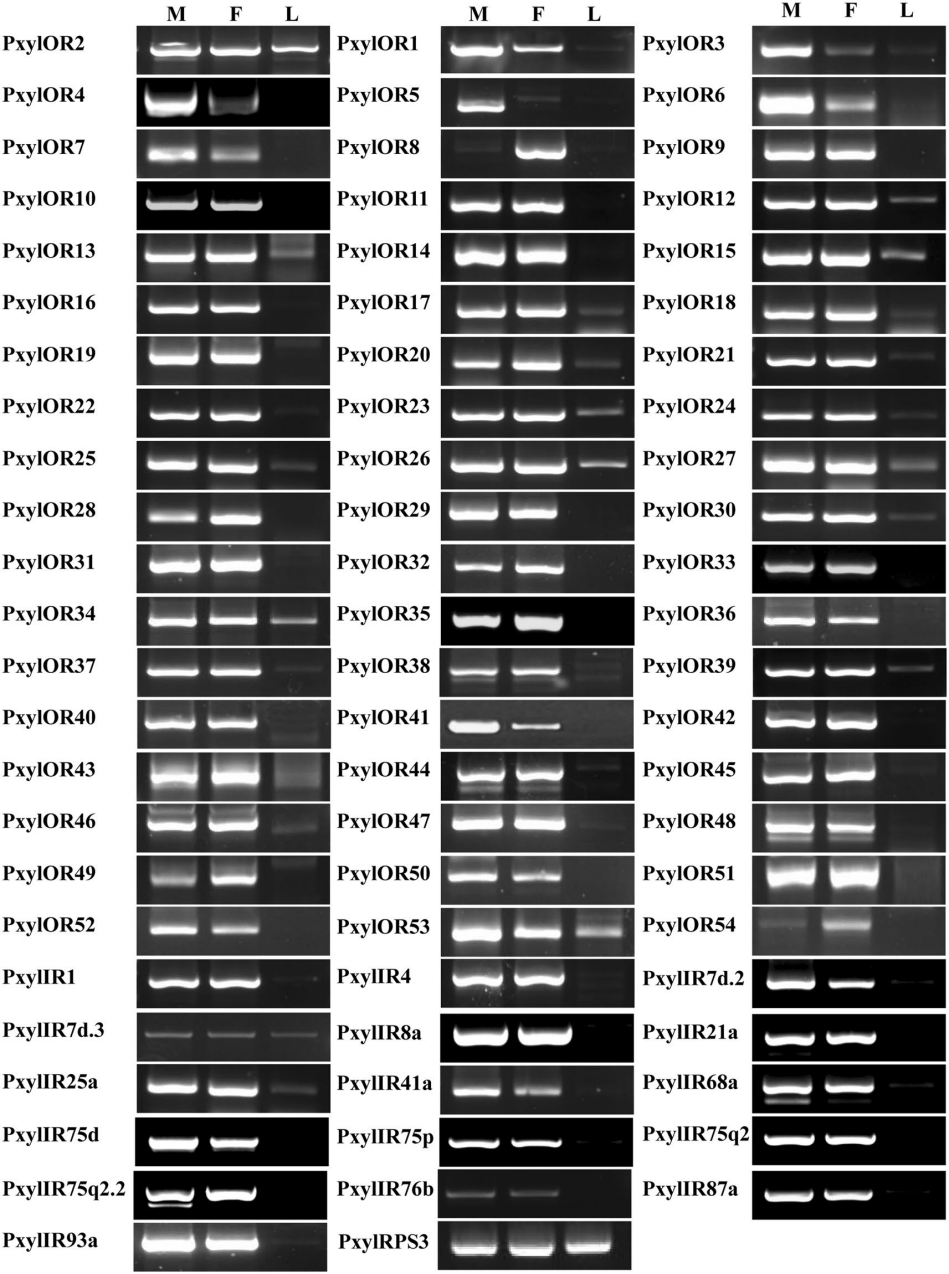
Tissue- and sex-specific expression patterns of candidate *PxylORs* and *PxylIRs*. M: male antennae, F: female antennae, L: legs. *PxylRPS3* is the reference.

To investigate the expression profile of PxylORs and PxylIRs between sexes and tissues, we determined the expression patterns of the 54 candidate ORs and 16 candidate IRs genes in the antennae and legs of male and female adult *P*. *xylostella* by semi-quantitative reverse transcription PCR (RT-PCR) (Fig. 5). As shown in Fig. 5, all of these 54 ORs were expressed in the antennae. *PxylOR2* was expressed in male and female antennae and legs. The expression of the nine candidate PRs was observed only in antennae but not in legs. And of which, seven candidate PRs (PxylOR1, 3, 4, 5, 6, 7 and 41) had male-biased or male-specific expression patterns. Different from the other lepidopteran ORs, *PxylOR8*, was only expressed in female antennae. *PxylOR45* was expressed in both male and female at a similar level. In other 44 general ORs *PxylOR54* expression was much higher in female than in male antenna and the remaining 43 ORs were expressed in both male and female antennae at a similar level. In contrast to ORs, the expression of all IRs did not differ significantly between males and females. All of these 16 *PxylIRs* were expressed in the male and female antennae, but *PxylIR7d*.3 and *PxylIR25a* were also expressed in legs.

## Discussion

In the present study, we profiled the antennal transcriptome of *P*. *xylostella* adults by RNA-seq technology and annotated 118 putative olfactory genes, including 54 putative ORs, 24 OBPs, 16 IRs, 15 CSPs, 7 GRs, and 2 SNMPs. Chemosensory genes have been identified in other Lepidopteran insects; 134 putative chemosensory unigenes were identified in the antennae of *H*. *armigera*, including 60 ORs, 34 OBPs, 19 IRs,18CSPs, 1 GR and 2 SNMPs, and 131 putative chemosensory unigenes were identified in *H*. *assulta* antennae, including 64 ORs, 19 IRs, 29 OBPs, 17 CSPs, and 2 SNMPs^33^. Our results are comparable with those from *H*. *armigera* and *H*. *assulta* in the number of genes identified. The identification of chemosensory genes from antennal transcriptomes was also reported for the moth *M*. *sexta* (91 genes, including 48 ORs, 18 OBPs, 21 CSPs and 4 IRs)^34^ and *B*. *mori* (138 genes, including 71 ORs, 20 OBPs, 16 CSPs and 31 IRs)^35–38^ and many other insect pests.

Insects utilize three groups of chemosensory receptors, ORs, IRs and GRs, to perform a variety of essential behaviors such as foraging, mating and oviposition. ORs are the centerpiece of peripheral olfactory reception and determine the sensitivity and specificity of odorant reception^3^. Due to the availability of insect genome databases and progress in sequencing technology, increasing numbers of *OR* genes have been identified from many Lepidopteran species. To date, 68, 64, 70 ORs have been identified in the genome databases of *B*. *mori* ^38^, *Danaus plexippus* ^39^ and *Helioconius Melpomene* ^40^, respectively. Recently, by using next-generation sequencing technology the antennal transcriptome of *M*. *sexta* was profiled, and 48 *OR* genes were identified^34,41^. In this study, we identified 54 ORs in the antennal transcriptome of adult *P*. *xylostella*. The number of ORs identified in this paper is less than that identified by You *et al*.^30^ in the genome database of *P*. *xylostella*. We might have missed some development-related *OR* genes because we only identified chemosensory genes in the adult antennae. Typical insect ORs are characterized by seven TMDs. We found less than seven TMDs in PxylORs, which is also observed in other Lepidopteran insects^33,42,43^. This is probably caused by the limited power of the software used for TMDs finding.

All of the PxylORs identified in the antennal transcriptome are highly divergent and share low similarity with other Lepidopteran insect ORs. A study showed that the common ancestor of Lepidopterans had fewer *OR* genes but that there were multiple gene gains and few gene losses during the evolution of Lepidoptera. This phenomenon of gene family expansion is suggested to be associated with the adaption of Lepidopteran species to host plants^44^. We also identified 9 (PxylOR1, PxylOR3-8, PxylOR41 and PxylOR45) candidate PRs based on their similarity to previously characterized PRs. The antennal expression pattern of PoxylPRs is consistent with that of PRs in *H*. *armigera* ^42^ and *S*. *littoralis* ^45^. Among these 9 candidate PRs, 7 showed male-biased expression, and PxylOR5 was only expressed in male antennae. In contrast, PoxylOR8 was only expressed in female antennae. Sex and tissue-specific expression of chemosensory genes is very common among Lepidoperan pests. It was found in *H*. *assulta* ^33^ and *H*. *armigera* ^42^ that some of their antennal *OR* genes showed sex-biased expression pattern. The male-specific expression of PxylOR5 probably plays a role in locating females, while the female-specific expression PxylOR8 likely also has ecological significance, i.e. optimization of pheromone production and spatial dispersion of females among host plants^46,47^ and selection of oviposition sites.

We identified one Orco unigene, named PxylOR2, which has high similarity to HarmOR2, BmorOR2 and HvirOR2. Orco is highly conserved among all insect species^3^ and carries out similar functions in different insects^48^ by forming a ligand-gated ion channel^49^. Orco probably functions as a chaperone and forms a dimer with the other ORs in *P*. *xylostella*.

GRs can respond to tastants such as sugars, bitter substances, CO_2_ and some contact pheromones^50^. Thus, GRs play very important roles in food selection and feeding behaviors in insects. The first insect GRs were identified in the fruit fly, *D*. *melanogaster* ^51^. The number of Lepidopteran GRs varies greatly; there is one GR in *Cydia pomonella* ^52^ and *H*. *armigera* ^42^, 2 in *M*. *sexta* ^34^, 3 in *Heliothis virescens* ^53^ and 5 in *Spodoptera littoralis* ^45,54^. In the antennal transcriptome of adult *P*. *xylostella* we identified 7 GRs, which is more than those in the Lepidopteran insects mentioned above, but far less than the number found in the silkworm *B*. *mori* (65 GRs)^55^ and the oriental tobacco budworm *H*. *assulta* (18 GRs)^56^. GRs are mainly expressed in gustatory organs such as the proboscis and maxillary palps, rather than in antennae^8^. This is a possible reason why we identified only 7GRs in *P*. *xylostella*. Two GR genes, GR21a and GR63a have been proved to be putative CO_2_ receptors in the antennae of the fruit fly^57,58^. And in mosquitos, 3 putative CO_2_ receptor genes (GR22, 23 and 24) have been identified in the maxillary palps of different species^59–61^. The PxylGR1 was closely related to the GR22 in mosquito and GR21a in the fruit fly and predicted to be a candidate CO_2_ receptor.

IRs belong to an ancient chemosensory receptor family, and two subfamilies of IRs have been identified recently, i.e. the conserved ‘antennal IRs’ and the species-specific ‘divergent IRs’^62^. The first IR was identified in the coeloconic sensilla of *Drosophila* ^14^ and most *Drosophila* IRs have clear orthologs within the genus of Lepidoptera^34,42,63^. IRs are ligand-gated ion channels that mediate chemical communication between neurons^14^. In this study, we identified 16 IRs in the antennal transcriptome of *P*. *xylostella* and named them based on homologous sequences from other insects. Similar numbers of IRs have been identified from other Lepidopteran insects: 19 IRs were identified in the antennal transcriptomes of *H*. *armigera* and *H*. *assult* ^33^, 15 IRs in *C*. *pomonella* ^52^, 20 IRs in *Chio suppresalis* ^43^, and 12 IRs in *S*. *litoralis* ^36^. All of these IRs are expressed in antennae, but PxylIR7d.3 and PxylIR25a are also expressed in legs, which is different from the expression patterns of these genes in *H*. *assulta* ^33^. Coincidently, HarmIR25a, HarmIR75d, HarmIR75p and HarmIR76p are also expressed in the cotton bollworm legs^42^. The function of leg-expressed IRs remains unknown and deserves in-depth investigation.

OBPs are believed to be directly involved in the activation of the ORx/Orco complex in the recognition of specific odors^20^. A total of 24 OBPs were identified in the antennal transcriptome of *P*. *xylostella*, including three GOBPs and three PBPs. The number of OBPs identified in the present study was comparable to those identified in transcriptomic analyses of *H*. *armigera* (34) and *H*. *assulta* (29)^33^, *S*. *litura* (21)^64^, *S*. *littoralis* (26)^54^, but fewer than those identified in *B*. *mori* (44)^37^. OBPs showed lineage-specific expansion and diversification; therefore, it is not surprising that there are some differences, or even big differences, in the number of OBPs. Previous studies have also shown that some insect OBPs and CSPs are expressed exclusively in non-antennae tissues or in larvae^65^. Therefore, different sampling and sequencing strategies may lead to different results. In a previous study, two GOBPs, GOBP1 and 2, were identified in *P*. *xylostella* antennae^66^. GOBPs were also found in the antennae of *C*. *pomonella* ^67^ and *S*. *litura* ^68^. The antennal *P*. *xylostella* GOBPs identified in this study have ecological significance, e.g. guiding *P*. *xylstella* to find better food^69^. The antennal *S*. *litura* GOBP1 can bind to plant odorants, while *S*. *litura* GOBP2 can bind to aldehyde-sex compounds and analogs^68^.

CSPs are a class of small soluble proteins expressed highly in the chemosensilla lymph^70^ and show high binding activity to odorants and pheromones^71^. We identified 15CSPs genes in the present study. The number of CSPs identified from *P*. *xylostella* was comparable to the number in *B*. *mori* (18)^72^, *H*. *armigera* (18) and *H*. *assulta* (17)^33^ and *S*. *litura* (18)^64^, but fewer than the number in *M*. *sexta* (21)^34^, *Sesamia inferens* (24)^63^ and *S*. *littoralis* (31) ^45^. Because CSPs are also expressed in tissues other than antennae^73,74^ and may participate in other physiological processes, it is possible that we have missed some CSPs in our antennal transcriptome analysis.

SNMPs are two-transmembrane domain proteins that share very high homology to members of the mammalian CD36 receptor family, which are thought to function in pheromone detection of Lepidopteran and Dipteran insects^31^. Two subtypes of SNMPs (SNMP1 and SNPM2) have been frequently identified in most insects, e.g. *Helicoverpa armigera* ^33,42^, *Cnaphalocrocis medinalis* ^27^, *S*. *exigu*a^75^, *S*. *litura* ^28^, *C*. *suppressalis* ^43^, *H*. *assulta* ^33^, and in this study, *P*. *xylostella*. The expression of antennal SNMPs in *P*. *xylostella* suggests their role in pheromone detection, similar to what has been reported in *D*. *melanogaster* ^32,76^.

## Conclusions

In summary, we identified 118 candidate olfactory genes that may function in odorant perception in the diamondback moth, *P*. *xylostella* by assembling and annotating transcriptomic sequence data. We carried out a comparative phylogenetic analysis to predict gene functions and examined the transcriptome patterns of the *P*. *xylostella* OR and IR genes. Genes with sex-biased and tissue-specific expression patterns, especially PxylOR5 and PxylOR8, are potential targets for environmentally-friendly management of this destructive insect pest. Our results lay the foundation for functional analysis of these receptors in both neurobiological and evolutionary studies.

## Materials and Methods

### Insect rearing

The laboratory-maintained *P*. *xylostella* was reared in the Institute of Plant Protection, Chinese Academy of Agricultural Sciences, Beijing, China. The larvae and adults were fed on Chinese cabbage and kept in cages at 27 ± 1 °C under a 16: 8 (L: D) photoperiod and 65 ± 5% relative humidity. Male and female larvae were distinguished at the last instar and placed in separate cages. Antennae of female or male adults were dissected at 1–3 days after adult emergence, immediately frozen in liquid nitrogen, and then stored at −70 °C until use.

### Total RNA extraction

The frozen antennae were transferred to a liquid nitrogen-cooled mortar and ground with a pestle. One mL of TRIzol reagent was pipetted to the homogenate (Invitrogen, Carlsbad, CA, USA) and total RNA was extracted following the manufacturer’s instructions. Total RNA was resuspended in RNAse-free H_2_O, and RNA quantity was determined with a Nanodrop ND-2000 spectrophotometer (NanoDrop products, Wilmington, DE, USA). RNA integrity was assessed using an Agilent 2100 BioAnalyzer (Agilent Technologies, Englewood, CO, USA).

### cDNA Library construction and Illumina sequencing

Tenμg of total RNA, extracted from approximately 2000 antennae of 1–3 day old adult male or female moths. The cDNA library for each sample was prepared using the NEBNext® mRNA Library Prep Reagent Set for Illumina (NEB, Ipswich, MA, USA) following the manufacturer’s instructions. Poly-A RNA for each sample was fragmented in fragmentation buffer to a length of 200 nt–700 nt. Random hexamers were used to generate first-strand cDNA, and second-strand cDNA was synthesized using RNaseH and DNA polymerase I. The double-strand cDNA (ds cDNA) samples were purified with the QIAquick PCR Purification Kit (Qiagen, Hilden, Germany) and eluted with EB buffer. The short fragments were treated with T4 DNA Polymerase and T4 Polynucleotide Kinase for end-repair and dA-tailing, then sequencing adaptors with barcodes were ligated to the dA tail of ds cDNA using T4 DNA ligase. To select insert length, ds cDNA samples were separated by agarose gel electrophoresis and bands of approximately 200 bp were excised and purified with the QIAquick Gel Extraction Kit (Qiagen, Hilden, Germany). Paired-end sequencing of the library was performed on the Illumina HiSeq™ 2000 platform (Illumina, San Diego, CA, USA) at the Beijing Genome Institute (Shenzhen, China). The read length of each end was 90 bp. The male and female libraries were sequenced in one lane, and raw reads were then sorted by barcode sequence.

### Unigene generation

Raw reads were pre-processed to remove low quality reads and reads containing adapter sequences and poly-A/T tails. The publicly available program Trinity was used to perform de novo assembly of clean reads to generate a set of transcripts^77^. The Trinity outputs were then clustered by TGICL (TGI Clustering tools)^78^. The final unigene dataset consists of uniformly clustered sequences and singletons.

### Gene identification and functional annotation

Unigene sequences were first searched against protein databases like nr, Swiss-Prot, KEGG and COG, using blastx with an e-value cut-off of 1e^−5^ ^79^. To identify more OR genes, 63ORs from *B*. *mori* were used as queries in tblastn searches of *P*. *xylostella* antennal unigenes. Unigene ESTs were predicted using ESTScan^80^. Signal peptides in the protein sequences were predicted using SignaIP 4.0^81^. The TMDs of annotated genes were predicted using TMHMM Server Version2.0 (http://www.cbs.dtu.dk/services/TMHMM).

### Phylogenetic analyses

Phylogenetic trees were constructed based on the amino sequences of the candidate olfaction genes and genes from the collected data sets. The OR datasets contained OR sequences identified from Lepidopteran insects (36from *H*. *armigera*, 18 from *H*. *virescens* and 63 from *B*. *mori*)^38,42,82,83^. The IR datasets contained IR sequences from *H*. *armigera* (11), *S*. *littoralis* (11), *Cydia pomonella* (10), *B*. *mori* (18) and *D*. *melanogaster* (64)^36,42,52,62^. The OBP datasets contained sequences from *H*. *armige*ra (26), *H*. *virescens* (17) and *B*. *mori* (34)^37,42^. The CSP data set contained sequences from *H*. *armigera* (13)^42^, *H*. *virescens* (9)^84^ and *B*. *mori* (16)^70^. All amino acid sequences were aligned using ClustalW2^85^. The unrooted neighbor-joining trees were constructed by the Jones-Taylor-Thornton(JTT) method with 1,000 bootstrap replications as implemented in MEGA5 software^86^.

### Expression analysis of the candidate receptors by semi-quantitative reverse transcription PCR

To illustrate and compare the expression patterns of candidate receptors in male and female antennae, semi-quantitative RT-PCR was performed using cDNA prepared from male antennae, female antennae and legs (male and female mixture). Legs were used as a control to confirm the antennae-enriched expression of candidate receptors. Total RNA was extracted as described above. Prior to cDNA synthesis, RNA was treated with DNase I (Fermentas, Vilnius, Lithuania) to remove trace amounts of genomic DNA. The cDNA was synthesized using the First Strand cDNA Synthesis Kit (Fermentas, Vilnius, Lithuania) and was used as a template in PCR reactions with gene-specific primers. The housekeeping gene RPS3 was used as a control^87^. Primers were designed using the Primer Premier 5 software (PREMIER Biosoft International), and the sequences are available in Supplementary Table S1. PCR was performed with the Veriti Thermal Cycler (Applied Biosystems, Carlsbad, CA, USA) under the following conditions: 94 °C for 2 min, 33 cycles of 94 °C for 30 s, 55–60 °C for 30 s, and 72 °C for 30 s, and 72 °C for 10 min. The cycle number was reduced to 27 and 30 for Actin and OR2 amplification because of their high expression level. The experiment was repeated three times using three independently isolated RNA samples. PCR amplification products were run on a 2% agarose gel and verified by DNA sequencing.

## Electronic supplementary material

Dataset 1

Supplementary Table S1
