# Supplementary material for: Identification of Genes Involved in Chemoreception in Plutella xyllostella by Antennal Transcriptome Analysis
Source: Sci Rep. 2017 Sep 20;7:11941. doi: 10.1038/s41598-017-11646-7 (PMC5607341; doi:10.1038/s41598-017-11646-7)
Supplement: Supplementary file 1 — Dataset 1 [file 41598_2017_11646_MOESM1_ESM.doc]

>PxylOR2

MMNKVKAQGLVSDLMPNIKLMQMAGHFLFNYHEENGGMSMLLRKIYASVHAFLIVIHYLC

MLLNMAQYSDDVNELTANTITVLFFAHTVIKLLYFAINSKSFYRTLAVWNQSNSHPLFTE

SDARYHQLALTKMRRLMYFICAVTVLSVISWVTLTFFGESVRFIPDKETNETLTEPAPRL

PLKAWYPFDAMSGGMYIVAFAYQVYWLLFAMAIANLMDVMFCSWLLFACEQLQHLKAIMK

PLMELSASLDTYRPNTAELFRANSADKEKVPDPVDMDIRGIYSTQHDFGMTLRGAGGRLQ

NFGGQQVNNPNGLTQKQEMLARSAIKYWVERHKHVVRLVASIGDTYGTALLFHMLVSTIT

LTLLAYQATKIDGLNVYAFSTIGYLSYTLGQVFHFCIFGNRLIEESSSVMEAAYSCQWYD

GSEEAKTFVQIVCQQCQKAMSISGAKFFTVSLDLFASVLGAVVTYFMVLVQLK

>PxylOR1

MRVFFLTDGSDLEGVEKLEDIKHIKVVKWTLTSLSSWPHPPHRRRRAYVEQFFLNLQSFL

CIPFIVIYLLRNTGKKDFFRMGHVWITFFMNIVASTRLLLPLTKNYQTLTKSFIEELHLF

YHRHTSEYSMKIHLEIHKLSHFFTMYLTGMMVGGIVLFNATPLANNIFSGAFKKDKPPDL

EFEHAVYYGLPFDSETKYSGYIPVFLYNWFISYFCSSCFCIYDLILSLLIFHLWGHLKIL

NYNMRTFPRPGCVAPHIKDTSRLRYDDEEMVIVGRMLREQIDYHRFISDFSDRMSATFGP

MLAIYYSFHQVSGCLLLLECSQLDADALIRYGPLTVILFQQLIQLSIVFELIGSSSEKLK

DSVYSMPWECLDDKNRKILLLFLKKVQTPIHLKAMGIADIGVQTMAGIIKTSLSYFAFLR

SK

>PxylOR3

MPAGAVYLDYVRILRRFLWFNGCWPGQLFGEEVPLFIRYHKYHVLGQFTVNLMAQINFLW

KFHKDISFLMMGHVYITTFLTSVSLVRSSLPHFEEYRNIVNTFLTEFHLYYHKQKGKYEA

EISAYWDKFSYWFSLCQMALMLLGMTSFNVLPIYKSIQAGAFTTRDIDRDNVEFAVYFAI

PGIDCYDYFYILTALNVYFSYITACSICVLDLLLSLIVFQIIGHIQILNYNILNIPMPEG

LKYNKEENSVIGKHLISIIDQHRYIVRFAATISSFFGPMLAMNYMFHLVSGCILLLECAR

PDPETLARFGPLTIIVFGQLIQLSIIFEFVGYISEKLIDAVYCMPWASMDVSNQKTVKFF

LSRIQTPIQLTAMGIVPVGVQTMLGILKTTLSYFALLKSISE

>PxylOR4

MKPGALSLDYVRTVRRFLWFSGAWPGEVFGEAVPRFIRCHKYLILVQYVLGLIGQVNFLW

KFNKEISFLMMGHVYITTFLTSVTLVRSLLPFFQEYKNITEEFLTEFHLYYHKTKGKYEA

EICAFWDWLSSWFALYQMALMVLGMTLFNALPVYKSIQAGAFTTTDLHNKSLEFAVYYTT

PLFHCYDHFYLATTLNLYVSYLTSCSVCAQDLLLSLMIFQIIGHIQILNYKIENIRTPEG

LKYNEDENMQVGKTLIAIIDHHRDIVRFAASISSFFGPLLAINYMFHLVSGCILLLECAR

PDPETLARFGPLTVIVFGELIQLSVIFEIVGYKSEKLIEAVYCMPWESMDVSNQKTVKFF

LSRIQTPIQLTAMGIVPVGVQTMLGILKTTLSYFALLKSISE

>PxylOR5

MSRKAGALDLQYIQILRRFLWFNGAWPGDVLGEPVPLLIRYHKYQIQVQSILVLIAQVNYLWKFNDEMSFLMMGHVYITTFLTAVTLVRCSLPFFQEYHNISKTFLTEFHLCYHKHKGKYEAEICAFWDWLSYWFALYQMALMVLGMTLFNALPVYKSIQAGAFRTMNLQNKTLEFAVYYTLPVYHCADHFYISTTMNVYFSYVTSCSVCVLDLLLSLIVFQIIGHIQILLYNIENISVAKGLKYNKEENMAVGKKLIEVIDHHRDIVRFAASISSFFGPMLAINYMFHLVSGCILLLECARPDPETLARFGPLTVIVFGELIQISVIFEIVGYISEKLIDAAYCMPWESMDVSNQKTVKFFLSRIQTPIQLTAMGIVPVGVQTMLGILKTTLSYFALLKSISE

>PxylOR6

MIQTGERSKALEVKYIKALSKFLWSIGAWPGEEFGDSVALPIRFQRLTLPYQCAGILAAQ

IYYLVNHRTNIRFFDVGHVIINCFLTLATGTRTALPSFKGYTLIVKKFINDFHLIHFKDK

GEYDEKVYKITDFVSYYFTIMQMTLMVCGMTLFNMSPLYNDYRMGAFSRHRPPNITMDFA

VFYEFPGATQDEHFYAATFLNLWLSWNCSVSVCSIDLLLSLMVFQIIGHIRILMYDFENL

ERPKSSESVKTEGEESLVPVTVELFDRQENMRVHRKLIDMVIRHRLIVEFADDISSFFGP

LLALTYSFHLVSLCILLLECSQNDPQALARFLPLTAIIFGELVQISVVFEVVGYMGEKLI

DSVYLSPWECMNVSNQKSMKFILSRIQLPLQVTAMGMVPVGVETMTAIIKTTMSFFAILQ

SIND

>PxylOR7

MNEKYLDLSYIRLLKKYLWYTGFWPGEALGEHIPLYIKWHRIQIVVQNVIGFVGQAWYIV

ENFTQMPFVVAGLLYIVASLTVLMAAKCSLTNFKGYQEIIRKLLQEFHLIHHSRGGAYEK

KIIAKVDKISHYCTVYHMAIIFFIVLTFNGIPIFNSYKAGAFRGRNLSGMKLELAIYFRY

PGFECLDYFYLLSFLNIYFTYIGAVAMFAIDAIVSAIIFQIIGHILILKHDIENLPEPKR

ETVVVFPHTGYEGHLGARVRLRLYDDEENKYIHNVLVGIIKHHKYILGYVNEVSGFFGPT

VGLNYMFHLVAGCVLLLECIRCDQDVLLRNLTLVGVSLVNLAVHSVTFEIVGSYSEQLID

AVYSMPWESMDISNQKCMKLFLSRVQTPIRVTTMGVIPVGIQTMGAILRTSCTYCTFLVT

VDEG

>PxylOR8

MEIKSACSGGALRLGYIRIVRRFLWFPGSWPGRALGERVPPLIRYHQYQIQVQTLLILVG

QILYLAEYHETMSFLTIGHVYITTFLAFLTSVRVLVPLLPGYKKVVKTFLMEFHLFYHQH

KGTYEKEIATYWDKYSYVLTLFQMAMVVLGMTLFNLLPVYKSYRAGAFTAWDLTNKTLEY

SIYYTLPGFNCLDHFYIATTLNLYLSYITSCSICTLDLLLILIVFQIIGHIQILKYNIEN

IPAPSRSVSIFVTEISPSSKSNVTVQLYNKEENYVIKQKLIGFISHHRFIISFADRVSSL

FGAMLAFNYLFQLVSGCVLLLECSSMDPDALARFGPLTCIVFGELIQISIIFEYIGYITE

KLIDAVYCMPWESMDVSNQKTVKFFLSRIQTPIQLTAMGIVPVGVQTMLKILKTTFSYFA

LLKSMNE

>PxylOR9

MFKPKEKSSTINNDQILNYTHYSELPLKLVGCWDWFPDAVDMKKIIANYVYLCLVIFVLI

NVTAMLMVSLYSEWVDIMSSLDMLADSLPYVASVLVVAYFAVYRAELYELNDFMNKNFKF

HSARGLTNMTMLKSYKMAKSFAYTYTVCSLCSSTMYAVVPMIIHWWTRTPVQGWFFMDVT

RSPFLEISFLRQILSQIFLGLALGQLGVFFASNSILLCGQLDLICCSARNARFTALLQNG

VKHASLVSQYHDILKDEEHNYIYNTSEMVDSIYHYDEKVTNDFSRIDFDIYSATHDAHTA

AALTAVARQGQVAAAYKRRFERFASPLLVLRVVQVTLYLCTLLYAASRKLDTMTVEYLLA

VSLDIFIYCYYGNQIIIQADRVSTAAYQSSWHTMGLKPRRLLLNILLANKRQMIVRAGGF

LPMDLRTYVNIIKTSFSYYTLLVNVNEHK

>PxylOR10

MGNSLRQSFNKHHFDFEAPVIHVHDFHPQIRVLLSMNGLFFNNKDSLLRFVLPAICICLS

VVATTLELLAMWHALQEQDSALFTEAFAYCVIIGVVPFLYLCNFMKSDKIYALVDGMDKD

FVTISKLGEPYRSNFLAGQLNIWKCGYAWLIFVIFVASMYILLSVVSILYYCLFVTHDEG

SHRPLVFPVWLPHDDPHRSPNYELFLGLMYLIIIDFATTFTTYVYIVFHLLLHCYFLINM

VMIGLEHLFDDVDDEVATLDCEDERYKEAKAVFKLRMKNIVDWHNAVFTLISNILAVYGT

VMAYQVIFSSMVMCLMAFYISVQLEQGNVDICITLLCVGALLQLWIPCYLSSLLRSKGYE

VCESAFYSGWHHAGLSRLVHADLRLVMLRAQKPVTIQVTMLPVLGLETFSSILSTAYSYF

NMLRQTSE

>PxylOR11

FNTQSSVKMQLLSSIWRKLTQTRALEYSSGSYETQFFETVYRVMFLAGVSSHDRGLRLAY

SYLVKLTLVVFVGSELWYLFSQTADLDHVIDNINVTLIHLIAMYRYRDMMRHKAIYKRLA

GAMESPHFDVSTPARRALLQAWARRSERYLQLLLALGTCTLAAWYVYPLVDDLDYNLTVA

VRLPFDYRTPQLYAATYLATLVAFNYTSYFVMVNDLIMQVHLMHLLCQYTVLADCFRNIL

EDCSDEEENKNENYHSLAWGDKYVKRLGDLVNQHKFIMANTLELKRIWSTPMLMQFLASS

MLICLAGYQVTATIKLSITKFLMSLLYLAYNMFELFIFCRWCDEIKIQSENIAEAVYCSG

WERGAAARGGVRARLMLVLTRARRPLVLSAGGLYDLSLASYSTLVKTSYSALTVLLRVSD

D

>PxylOR12

ILSRSKGKSVMDKSTLKKNFHTEITFLNYVASKLFLHPRTEGKGNNRNFYGYHLIYSLVW

LAIIQLSITLYLYGLKNLIAFTTVAPNVGVCMISALKYSIIYNNKPYYDRFFKHYGEDIW

QTIPESKENAKVISKYTFISKVINRVFVCYSLPLVLYVDSFPWLIMKFQTKFLGKEKQLL

YPFDGWYPFDKTVWYFAAYSWESLMTGIVVMIYMYSDMINIFSVTSICMEFRILGISLKN

LVSDEDIQQMKGKDAEQVNRRIKNDLKTILAKHDVLAGMCKELDQLLGNTMFANYTSGCG

FICLTAFTFTVVDDFYQSIRCFFFFLSLLAAVLDHCIIGQIISDHSMQLADAIYSSNWTH

ADQSTKRTLLILLMRTQKPFELTAKGFVTMDLNTFTDIMSTSYQFFNLLRTCYLPQMGEI

>PxylOR13

ICDSGFGAIWPCLFKMSTYRQIDCFNINVKYWKFLGIYPRRDTTRLYEYYSFLFISFFFV

FYISLTTLNFWFIPGPMDLFIEETIFYTTEVAVLAKVFTFVCMKDKVVKLLDILESDMFQ

PDTNHRIKIVKDAKKFNITYWKVMAAISYLSNSTHVLSPLFTHLLTGADLVLPLCSYLFL

ADDFRNAYIYYCYTYQSIGMHFFMLVNVNVDSFIQGLIILAIAQLDLLDDKLRKVTENNN

IDKDSRNESDTNEDIDYVRKLNKCIIHFDNVAKYCGLIEEVFSLVLFVQFSIASVILCVC

LFRFTLPAPREYYIFLVTYVFVMVGQILVPCWFGTRVIYKSSQLSLAVYDSDWTPRSRRF

KSNLRLLVERLNRPLTIIGGKMFPLSLVTFTSIMNSAYSFFTLLRHMQSREDEAN

>PxylOR14

MNFFKRKSAEDVFAIRETDGVDEQKRFKPFLETYRIVAFTMIIGLIFPNLNTVKKRLRIL

VFLYVLAAPVASTVVLDCYMSLRRMDLINVTRHCTIFIPFFVVLIKMYLMNVNRYKAKEI

IDTINSDHARYNTLSEDYQDIVTDNIKATVVFEKTWAFCVIGCVLMFPLMAGVQTLYSQL

FDEIPKKYMIHDTNKPFSEPEARFESPFFEIMFVYMFVMALFYWVNFTGYDGMFGVAVFH

ACLKMKMYCHSLKKAFEETSDVEQLRRRISDVIQEQVEVYAFVDKIQETFNTWLGIILMG

TITQICNCMYQIIEGDFDLRYLVFCCGTIVHIFLPCRNASELKYMSTETSTLIYLCGWET

VTDKSTRRSIMFMIARGQIPLEITAFNMFAFEMELFLSILQTSYSMFTLLRD

>PxylOR15

IFSCHLTVIILPAMASQPISTAFDYNVRCWKILALWLREVNHKYYYYYSLLFIFLFFVLY

DGLLTLNLMYCPKTVQAFVPELLFYFTSVVVAFKIHMIISKSKCILEAFSYLDTDVFAGE

TPEHKEMNNTFVADYKRYYLIYFCIGHISYIGFTFYPLINHLVFGEKLVLSVCNYYFLTE

KERDDNIYLYWIFQSLGNYAHMTTSVNSDTFMPGLIFMGIGQFKALNYRLANIRSLSQEG

NLDIRQERQLEEELVKSLRHYDAIRVYCKLIQEIFDMAMFVQFGVGAAINCVTFIACLQI

PKNELFFLVLYGVMMTVQIFTPGYVGTQIAHESGLTTSAVYDCDWLERSASFKRNMILLV

ERANKNMILTARSMFPLSLTTFISIMKAAYSCFTLVRAVNNQETGAYLP

>PxylOR16

MSVDPTVQEQAKAEILQSLNLSIFSMRQFGLSFDKPPNRRAFIKQKLILYLCFFGISYHI

FSDIVNIGVTLATTPRVEFVVPLFHTFGYGALSSFKLWSVWYKKDVFEQRIADLVDIWPV

PPLAPELQAIKDKSLLALRIAHRFFFGLNVSAVWIFNLTPVMIYVYESWWQGRPDAVVGF

PWTCWYPFDKWDPTNHVFVYLFEILSGVTCVWAMSASDLMLTGMASHICMLLRILHQRLT

SLAASEQPPPDHYRDIVSCIKLHQRLIVYCNDLEEAFSIVNLVNIVLSSINICCVVFVIV

LLEPLSALSNKMFLGAALIQVGVICWYADDIYHANSAVAAAAYSCQWHKTSPSCQRALMF

LIKRSQKPIALTAMNFTNINLTTFSSILYKSYSYFALLYTMYKEN

>PxylOR17

MEITVNNEKKQKYQNFNGTFRYCTFALAIALIYPNPSRRVKKIKFMVFCLIILSVSFVLL

WFISYLYFCYIARDMFNFTRNMTVGIVIFLFFFKTFYLNYKSDGFGVVMDAISDDLVKAN

DMDEVYQEIFDTYIKKGLVAQMIWIFIPIVQTCIFPVHAGLTMFFNLINEVPQPRVMVLE

MDILMVRSKQMESPWFEIVWFYTILGGFVLFPNFIGFDGSYCISVNHLCLKLRVLTEKLR

RAFAETTTDEQLEKRVKEVIKEHQASLVYYNLLQDVFGGWMFVVFFITSLEITFNLYQLS

LGGMDPKYLIFAFSTVVHNFVPCYFCTRLIEHGDDFCEALYMMPWEARHCQSVTRALAFM

IARTQGPLFLTGMGMVTFNMELFVSVMQSSYSFFTLIRD

>PxylOR18

MARETLSDTFNHNKLFWRITGIWFRQVKNKRFKYYAIPLIAIVFVAYDIFLTLNLVYTPK

KLETFMPELIFYFCEVQNAFKVYMVIFKSHQIVQAFNMMDSDIFIGESEEHKRITIKAKL

LFIKAFKVYFWLCNAGFACNSIVPFISFLIFRTKLYLPVCNYYFLSDETRDHYFWYILTY

QTYCVYNHMMYNISIDTFMSGLILMGLAQFRALNDSLKNIKCVSKNKITDEAEERRLRTE

LVKCLKHYDYLREYCSLIEEIYDPAMFVQISVGAASNCVILASLLLSMSSNDKSFIVLYG

SVMALEILMPGYLGSQLTYESEELVRSVYECDWIERPESFKRILKLLVERAKQPIILTTW

YIVPLSLNTFISIMKTAYSSFTILRAVSTRNAEESS

>PxylOR19

MFEKLLSKLEDPNYPLLGPNIKCLKFWGLLLPEEKTFMRKFYICVHSCMFCFMITEFIDI

WYIGSDMNQLITNMKSTMLAIVSVNKVVTYLWWQKDWKNIMAYVTKADIEARTSTDEENR

EIITVYTRYCRRISYLYWLLTYTTAAAVIALPMSYWFSSSTYRDNVRDGTEDYYQVVSSW

VPFNKNKLGGYLAASAVQSFATTYCGGWISSYDTNAIVIMVFFKAELQLIKNRCSKIFEV

DDEEEIMNRIKECHRRHNVLVKYVKQFDSCLSPVMFLYMIACSVMLCSSLYQTTSQASFT

QKLLTTPYLLLGVSQLFMYSWHGNEVSFMSNELIRGIYESGWWKKATVRREIILLVGTLD

RPIEFTAGPFFNLTIAVFVRILKGAYSYYMIIIKK

>PxylOR20

MPPETISSAFYSNVRGWKIFALWIREVNNKYYKCYAALSLVFLYFLYDGLLTLNLIYSPK

NLNTFIPELVFLLTSFVLVFKINMVMFKSKLILRAFSIMDSDIFSGETVEQKRITKRYVE

MYKKYYRAYFVLGYMSYTFFTIVPFLKHVFFGQELILAICNYYFLSDKERDANIYLYWCY

QSLGNFAHMNYGVNIDTFIPGLIMIGIGQFKALNVRLSNIKTYSKKNLVDIEQEKKLEEE

LIKCLKHYDLLREYCSLIEEIFDTAMFVQFGVGAAINCVTLVAMLQLPSNEMFFLILYGV

IMAIEIFTPGYLGTQLEHESERTTSAVYECDWMDRSPRFKRNMMLLVERANTSVELTALT

MFPLSLETFVSIMKGAYSCFTLVRAMNGREDTK

>PxylOR21

MKSFTAWVGAYFTDPNYPSMGLSIVYLKVIRLWKLKKVDYILPCWLCIAFLSQIAYILVT

NRRLHFIINMFQTGFFHLGLAKIALYYYNLPKWMETFNWLSEMEIRQQNDEHLKPIVDRY

TKYGNSVSWAYLILSYSTWVFNYGENLLMVIIQMETLHEIDPTYITFFWLWPTEPLKGRW

NVYPYIILQFFYAFLTCTYLLAFNVLCVSTMIAMAGQIEALSEMFRRALDTDSEEDQYRN

LINCYKRYADILFTQKRLNKIMSPILFMYLLIASINMSLILFSLANLKKSSKIASQVLVV

SLVVEAFYYYWHGHQVMHQSENISAAVYDSDWVDKSPKIRRLVYIMSSTVNRKLVYNAGP

FNEVTVVTFIQIVKVTISFYRLMCTTTLSDSGP

>PxylOR22

MKAFVASVAAYFTDPKYPSMGLAIVFLKSCGLWKLMKVHYIIPFCLFITFMSQILYMLFS

KRIAHFFLNLFQTGFFHLGLIKMALYFYNLPKWMETFNWLSETELQQQQDEHLKGIVKRS

TKYCNTVAWAWVIGSYSTWTFNYGENLLMVIYQIETLHEIDPTYITFYLLWPTEPATGRW

NVYPYIIVQFLYSFFTVTYLTVFDILCVSTMIAMAGQLEALSEMFRRALDTDSEEDQYRN

LINCYKRYADILFTQKRLNKIMSPILFMYLLIASINMSLILFSLANLKKSSKIASQVLVV

SLVVEAFYYYWHGHQVMHQSENISAAVYDSDWVDKSPKIRRLVYIMSSTVNRKLVYNAGP

FNEVTVVTFIQIVKVTISFYRLMCTTTLSDSGP

>PxylOR23

MAAEHLSRAFDQNVRGWKVLALWIREVDNKYYKCYAVLSLLICFVIYDGLLSLNLLYCPR

NLEDFVPEVLFYFTSVVLVFKIHMVVFKNKLILKVFSILDSDEFAGETAEHKEINATFIS

LYRKAFVMYFWIGHIAFMGYTLIPLLKFIFIGKKLTLSVCKYYFLTDEEKEDKLFFYWCY

QSIGNYVHMNYSVHTDTFMPGLIFMGIGQFKALNMRLSNIKSKSKKGKDLRHEKHLEKEL

VQCLKHYDMIREYCSLIEELFDSAMFVQFGVGALINCVAFIGLLQQPTEGRFFMMLYDVM

MTVQIFTPGFAGTQLAHESGLTTSAAYSCDWISRSASFKRSLVLLVERANQAVELTACSM

FPLSLATFISIMKGAYSCFTLVRAMNIREENE

>PxylOR24

MNRLVNSIDNYFTHPQYPNLSVAIVYLRMIKLWEPRKIFYAIPVTMGITFVSQILYVLMS

DQPFRYFLNLFQTGFFHLGLAKVVLFFYNHEKWLNIINWLSELERIQLHDPFLRPVVLRY

IRYGRILHAVMYPFGFIVFALNYGENYVMVAMQMESKGEIDPTYITFFWLWPTEPLKGKL

IVYPYVTLQWFYAFGGVCYLMAFNITCGSVVIGLGGQLEVLCEMFRRALDTDVKEEQERN

LIRCYKRYIDLQDTHNTLDAIMSPVLFMYLLVASINMGMILYSLPTLEKSSMYTSMVLVS

SLVVEAFYFFWHGHETMYQSEIVSAAVYGCDWVDKSPRIRRLVYVMSATTNRVLVFHAGP

FNEVTVITFISIVKVTYSFYKLMTTTVTHGS

>PxylOR25

MKWFTTYIDEYFTDPEFPSLSFPIVYVKLLKLWDAPRVYYAIPWSLALALVSQVIYIPFS

GRPLHFFVNMFQTGFFHLGLTKLAFFFWNWDRWNKIIYWLSGLEREQLRDEQLKTIVSSY

IKYNKRTTWYFWLCGMGTFFVNYGENFISVIVQMETTGVVDPTYVSFFWLWPTEPMTGGR

LNVYLYLGFQFIYAWGGVSYLLVFDSMSVSVMMTLAGQLEVLRDMFRTALDEDTEEKQHE

SLIKCYKRYIDILDTHKLSNELMSPVLFLYLLVASINMSLILFSLPKLGLSATITSVELV

ASLIVEAFYYYWHGQQVLYQSEIISAAVYECDWVDKSAAIRRLVYIMSSTTDKRLIYQAG

PFNDVTVATFISCVKVTYSFYKLMTTTVS

>PxylOR26

KMIRRFLKSLEDPQYPLLAPNLWMLKKIGMILPEDKTAKICYLILHEIVTFFVITQYIEL

YVIRSDMDLVLTNLKISMLSIVCIVKSNTCVVWQKNWVEVINYITTTDKEERDDINPQRS

SVLKSYTKYCRKITYFYWVLVFITFITVITTPFIKHLSETYIENIDPSELSDNFEHIFSS

WVPFNKNEYPGSWITIAWHVFVCAYGAGTMAAFDVSAMVMMVFFGGKIDLLRLRCQGIFG

NMEKGLSDEETSEMFLRLHRAHVLLLRYSRMFNSMLSPVMFLYVVMCSLMLCASAFQLTS

ARSATQKLLMAEYLVFGTVQLFMYCWHSNDVLYKSERVTLGPYESDWWKTSAKQQRSLLL

LTGQLNKTIFFTAGPFTYLTLPTFI

>PxylOR27

MEPSKVLVSPGHKYFEFNLKFLFAVGLWPHKEWCRNKLELYKMYETNLHILSVVYLIISS

IGTYNIRGNMEILMSNLDKSLIGYVFVFKIFAFEIKRKELRSLVNDIMQSGDKITKKCED

RMTSLLMFVMILVTMIVSAFSMSALYDGEMTVEAWMPFDPLQSKKHLLMAAQILAVTFMP

CALRGVGLQGIVCSVIMYFCEQLQDVQMKIRHLHYSPHRDEEVRQEFKDIVKKHVRLIRY

AKSIENVFNSFMLFHNLAMSVELCLNALMISVVGTEEKKTLVNFLAFFGIALLNTYILCY

LGNEMIIQSEGISQAAYEASWSFWPIDMQRDLLTLITVSQRPLKLSAGGIAVISLQTYCQ

ILYNGYSIFTMLHDMM

>PxylOR28

RLHRVYMGFVIFLPYLYVAQELAYVYTVRGNPDQVIYGLFKLFSYIDFIYKKMVLLLKAD

RVEKLLNTMKGPLFNQEGLHHREILLTQVASAKSTLYIFNFMGNLSCLVWLLVPIVQYSR

GHEIRFPLLFPVEINKAPNFYIIMVYLSIQWVNHAYINSTLDVFMSCLLGQCTAQIRILR

LDLETAVARSHEKAHQESISFSEAFHKVFTVSLLHHKEIVRMAVNIEDIFGGAMFCQIVV

TGWIFCTTAYRAVDMNPASVEFCSMITYITCVMVELVLYCLFGNRLAIESDKISNSAYSM

DWLQLPVTHRRSLLIFLERVRTPIQVVAGYIIPLSASTLVQVLKSSYTFYAVLNKSKEI

>PxylOR29

CLSSKMGFVGLTYMMPCIVYGILGFVKIANILHKVDIISKLIKELHAMFPGNGDYKESED

IDEEAESDIVIDSLKLSRKVVFVLFWTNLLLILLFDLRPLIVLAFKYFAHGQLELSLPMF

MNYPFDPYDLRFWPIAYGHQCYSSACCIYNLIGPDTLFFVCCTHLYMQFRILKRRLEIFI

TGSEIGGELEVAKKFSILAKRHQKLIELVGRLERVYSTSIFLNYGCSSFLICFTGFNVTT

LNDAWSVLNYLVFFSSNVSQIFLICLFGDLIMNSSREVADGIYNCKWYATTPKIRRSILF

YLVRSQKPCKLTAINFADVNLISFTTILSRSWSYFALLRTVMSTRSQDQQTLAAIA

>PxylOR30

WMETFNWLSEMELQQLEDEHLKPIVDKFTKYGNRVTWGWLIASYATWVFNYGENLLMVIY

QIETLHEIDPTYITFYLLWPTEPATGRWNVYPYIVVQFLYSFFTVTYLNLFDVLCVSTMI

AMAGQIEVLSEMFRRALDTDSEEEQYRNLITCYKRYADILFTQKKLNSVMSPVLFMYLLI

ASINMSFILFSLANLQKSSKIASQVLVLALMIEAFYYYWHGHQVRYQSENISAAVYDSDW

VDKSPEIRRLVYIMSSTVNRKLVYNAGPFNEVTVVTFIQIVKVTISFYRLMCTTTLSDSG

P

>PxylOR31

DFYRWSRVVFWVCLSMPITTMVMWSFLSRLPTHRGGNAVEFGIWLPFDPNDDAYFTLVML

YEFTQLTWLGCNNCNVDTFVTILLQQCTTQIRILRLDLETAVERAQENAESEGITFDEAF

HKVFTLSLRHYNEIIRLSKNIGHIFGRPIFFQFLVTAWIICTIVYRIVDIDPTSVLFISM

IAYMSCIVVELFLYCYFGTILTYESMKLTNAAYYMDWIRLPSRHRRALVIFMERVKSPIE

LLAGNIVPQSTNTFVSIMKSSYTFYAVLKSTNQIDAASP

>PxylOR32

AKAAIKHFDSIMQLFLFLCVSVFTFFCLLPLLLMVYQRYTTGVAEVQFPFSVKYFFDIDV

NHSSIWPVVYFHQVVSTLIVSLNVYGSDSLFYACCNFIQMHFRILQCQIEFLIGRQWGPN

DSHTLNEKFKGIVRRHQGLTEIVRQLEFLYSYSSLFNFVTSSFLICLSGFIVTTSKETRL

VLCFVTFLFMSLAQISIFCYGGDLIMQSSKDVSAAVYRSRWFVADERLKRSMFIVLARAQ

RPCKLTAANFADLNLSAFATVLRRSWSYFALLKTMND

>PxylOR33

TIKLAYLVSKTYSLIVLVTASMWGVFPLLKANTSTLTLPLGYPYVSQQSSIMFTVMYVYQ

LSSIVLNGFGNTAVDFLAGGLTMLASAQLDLLSCELGELGGGGGIGGSNGDNIDYSYRYA

VNCIEYHHKIIRFVKEIENIFSVPVFLQFALACMVVCMTAFKIAGTNETTQIIRLMFYLA

TILMELLVYCYFGHVIISKSQKVAEAVYSSDWQRTSLKTQRSLMLLMLRAQRPLSITAGK

RFHLSLVTFVAILRSSYSYYAVLNHHMKNSSP

>PxylOR34

MVPGTDTPENNAFLSMALMAEKQMRKAGLWQGAARDRTHWLAHCLLFILLTLSGLQVIKL

FISFDDLGTLIDCISVLSFVSMGLLKVVSLWLNVTKWHTLFSKITEMEENSITDLEYEMP

QQTGEKQSRDYTNTIQRILRYLPRIYWCAGGVFILSPFAELGLKYFRHLPLEWPHILTIW

VPYEDNAYVNVFLILIELFAAVYVIIVHIAFDVSSLAVMIFMSGQFAKLRWNTEMIGGGE

LSESSVSRDDKAHALIITCHQHYLDLTRLN

>PxylOR35

FFVGCNMSYICHNFLPLISFLVFGTELNLPVCNYYFLSDESRKHYFWWILVYQTYGIYHH

MMYNLSIDTFMSGLILVGLVQCRALNHGLQNIKSCAPKKENDTEDEKRLRMELVKCIQHY

DSIRKYCAHIQDLYDPAMFVQMGVGAASNCVILASLLLSMPSHEKALYVSVLMYGTVMAI

EILMPGYLGSQLTHESGQIVRSVYSCDWIDRPESFKSILKLLLERAKRPIILTTGYIVPL

SLNTFISIMKTAYSSFTILRAVHTRNMVE

>PxylOR36

IYRMLVREDQSLRILAFEMWFPWSLEDMRVYAASFIFHAYAGLICCSGYPGFQMTIILLV

GQSIRQLRILTFVISHQDELAKEITNMREGESLWQLYCTDILSQCIKHYITLKTFNNRVN

VIFRPFYLTLILVATLLVCGCSTKIAISNKFALEVIKYYVHEFCFIIVVLMFCLLGQQLQ

DECEKLEGSILQKWYIYNAKHTKHLRIFKMAASQRMPIHIFGTITLSLPTFTWFIRTGTS

FFTLMMSFMEEQ

>PxylOR37

ARQVGLFFTDDPPSNLLQKLKNKAAFVLSAAALLLLVAGELAYVLSLLAANTSVAEFVAS

LHIAGYGFISVGKMAIIWYKQDIFKQLVRELADLWPVRKDDGEAAEIKQRSLNALRFFHI

FYTAFTVLGVALYNLTPIALHAWRSARGARSELGFVWHVAYPFDKRHPVYHKIAYAFEVC

SGVSSVFFMLSSDLLFTATTSHITMLLRLLQLKIRRLAPERGAAGGEAGGRGAGG

>PxylOR38

WPTGLYSGRWKVYSYVVWQLFYGICSNIYVNGWDGLCVAMMILLAGQVEVLCEMFKRALD

TDDEEEQEKNLVDCYKRYVELLKAHNLCNSMMSPVLFVYLLFASINLALILYSVDSLETS

GVFLAATLFGGLMVQLFFYYWHGHQIIYQSSVVSASVYASGWVAASARVRRRVFLMSATT

DRRLVFHAGPFNEVSLTTFVAIVKTSYSFYKLMSS

>PxylOR39

VKSKDKTMTIDIEALYLKLPKNIMTFFGVWLPPKRHIILHNIYMLLVMVSQYSFLLFEFI

YLFDVLDDLEAASEASYLLFTQASLCYKTTVFLVNKRHLIELLELMRCEMFAPETEVHEK

FLSVLAVRIRRLCLFFMTSALTTCTLWAMIPLFDNQGRRSFPFRIWMPVTPERSPQYQLG

YLYQVAAIYISAFLFIAVDSVAVC

>PxylOR40

TFYHIYSAISFMFLLGTYLIIQVVDMCLIWGDLQLMTGTAFVLFTNLAQAAKLVNLVLRR

RRLRAMLADADAVLRGAAGEEARRIVERCDRGTSLQQLLYFCLTTVTVAGWAGSAEKNQL

PLRAWYPYDTSKSPAYELTYCHQVGALFIAAYLNVAKDTLVSTLIAQTRCRLQLLTLALS

KLHDGMAAGSATLLSPAEES

>PxylOR41

VRSSLPHFREYRNIIKIFLTEFHLCYHKHKGKYEAKICAFWDWLSYWFALYQMALMVLGM

TLFNALPVYKSIQAGAFTTTDLHNKSLEFAVYYTTPLFHCYDHFYLATTLNLYVSYLTSC

SVCAQDLLLSLMIFQIIGHIQILNNSIENIPIAKGLKYNMEENTEVGMKLIAIIDHHREI

VRFAANISSFFGP

>PxylOR42

TITLSTQYLFLLFQIIDIIRVFGDLEAVSQASYILFTQACLVFKITLFLATKNSLRLLLE

QMNSQVFMPQSTEQERILKLQALKIKRLLLAFAISSQATCGMWALKPLFDDAGEREFPFR

MWMPVGTEKSLEYIAGYAFQLLSVCTSAYIYFGVDSVALCMVIFGCAQLDVIKDKIVKIK

PVTMEDRAAKRAL

>PxylOR43

GLFLMGIAQFKALNSSLINIKETSLDHLNCMEKDDHLHKQLLKCLKHYDLLLQYCSLIQE

LCNPAMFVQFGIGAASNCVTLAALLLPLPTYEQIFIVFYGSVMAIEIFMPGYLGSQLRHE

SEQVIRAVYQSDWIDRSETFKRTLKLLVERAQKPVIMTAYSIISLSLSTFISIMKTAYSS

YTLLRAVHTRNN

>PxylOR44

RRLIYLLIISISFWPVYVMWFYDVFHCLQRLDVDNLTRQLSLGVPIMFCQFKMFLVTILR

KKMQNLIEEINADYERYNCMDAGYQAIVMENQKSLLKLEKVWTYMVLATVAAFPLMALYQ

TTEHHLFKEGQRFMVHDVDVPWLKEQRFESPYFEILFVYMVYIALVLVLCYTAYDGMFFL

CIFHACLKMK

>PxylOR45

IAYAYINFDHMASSQIEAMLIMIGLNILVVTKVFSSKLEAYTTVMKNFMNEIHLFHHQTD

EYSKQKLISREKYTRWTAGTLAVCISADIILYCAVPIYHNIRNKEFIANKTAILQTSIYL

VMPFDYGYNYKNWLIMHCINTYSTTVAGYLLIVFDVVNYAIVFHVIGHIEILKHKMAVFS

DAVKSLNER

>PxylOR46

DLWFVVLMLYFCKASEVVVKILAVDRIQPDETPLAYAERLDRSLRKFYKNHIKLVKFTLK

LSEMWRWMALIPLMNAAMSICNILLVLSHGIDLKFVPHIFPLFCEVFVYSYFGEQLKSKM

IEIRTALLEFDWTSLVKKNRQHYYIIVLYVSKEFGLKTAVGHDLSLVTMSTVLKSSYQAF

TVMNTVK

>PxylOR47

YQVRSDTDKVIDSMYLFLSFVASVYKQVILWLYADEVEGLMDIVKGPLFNREDERQKTIL

RDLARRAKFIHNWAFGLTQVTCVLWTLKSMVMHLSGSEVDFAIWLPFDHNHRTYFYFVLF

YYWLVTSWLGCNDCNADLFASALLAQCMYQLSYLRIDLETLVETSKEKALKEGRVFGDVF

Q

>PxylOR48

VVVCVLVFMTLLLTFDFKHITEEASNDGREWRINLIYLPFFIMYLFKFLLELQFILAVTH

VNSSLRAINHALEEELHSLDTRTEYVDHPIKYINKLIDSMAVTETSKYTVKLSKSNNYSW

LRHLARCHGCVSDATLALNDANGPILFFIVVNLLLHLINTPYYLITDILKFEGVKISFLF

>PxylOR49

VVIPIMEKLSYKSLSPHVRMLQVSGILPLDRDAPAWKYRLHQAYTALMTVIPYLYALQNL

GHMYKVRHDAVQVMDSMFLLLSYINMIYKKMVLLLDADQVYELLHVMKGPLFNQDDAAHR

EILANHAARALSSLKLFHYLALCSCFFWMAFPTFEHFRGYDLYFPLWMPIDPNKPVK

>PxylOR50

AYVPFDKYSPRGRLIANIWFAMGALYGCAVFTAFDVAAVVVMVFLGCKLQLLAARCERLF

APAPGGDRFWETVREVHTEHVMLIKYSKIFNSLISPVMFLYTLLCSLMLCTTAFQITSMP

MNITEKILTVEYLVFGVSQVFMYSWISNDVLCKSQLAMQGVYHSAWTDGLGAGARAG

>PxylOR51

AYPEIMSSWVPFDKTRPPGYWLYVLEHAVICVYGGGIVATYDANAVAIMTFFAGQLRLLR

ADCAAIFGDDARPASDRQALQNIRDCHQQHLFLIKYSRLFDSLLSPVMFLYIIICSLMIC

CSAIQLTKAGTTRMEQVWITEYLLALVAQLFLYCWHGSRALHASMEVEGGVYLA

>PxylOR52

LVKQLVLVLDADRVDRLIQSLDDPLFNLGDARSTLQLQATTRGAARLARLYVGTAVVTCV

LWIVFPILDHVKGNPVEFPLYIYVDTNRTPTFIVVLIYTYLVTSLVGIANTSMDAFMGTI

LLQSKTQLSILRSNLENLFESAEAESAGGGGARGERRPSTVVAPRAGVIG

>PxylOR53

LRSMDETLQCLNRVLSFVGISIFAKNNWDSPFYRVIQIFNLIIAIITFILTTGCIINSFS

DLPALIESICIWTTGVILSISITICWVFRKRFRLFVLEMGFRDTILDVPLIHYVMGLESG

GELLMELKELVVDSRERLLRFVRVLLKCYVSSVFVTASLYIVGA

>PxylOR54

KRTSLRVVITLLNKNDSLVTGSDERRRRHATMIRNIKNIILIFYGGIYLSEIFNYLPNRV

RVTQDYAMVPCVGIIRDPTASPNREICMAMLFVQECAMMVSVLNYQALLIVLIAHTAAMY

ELLADELLELNHQAPDLLADELLELNHQGPDTTSDTEQSFQTY

>PxylGR1

MGVMPIMRVSRHAKTTKRTTFNWISKATLWAYLVWSLECIVVVKVGRERLETFQNSSNKRFDEVIYNIIFLSILIPHFLLPIASWRHGSQVAIFKNMWTHYQLKYLKITGTPIVFPNLYSLTWGLCIFSWGLSFAVILSQHYLQDDFELWHSFAYYHIIAMLDGFCSLWYINCNAFGTASKGLATNLHKALEADHPALKLAQYRHLWVDLSHMMQQLGRAYSNMYGIYCMVIFFTTTISLYGALSEILEHGLSYKEMGLFVIVGYCMTLLFIICNEAYHATRKVGHEFQVRLLNVNLGSIDRNTQREVEMFLVAIAKNPPIMNLDGFANINRELFTANISFMSTYLIVLMQFKLTLLRQGARKMLKTIVMAVFNSTTTLADDDEEEEPDVGQ

>PxylGR2

MLARFKKNTKEKSEQNVVEAVSMSATVFINAWGSCVLYRRCLLGRVTRHFSTIGFLVWILRLVTFFYLTYIVFKEDQSIVRSFVTTKLHHYGDHYESITAIVFITYLFWKLPFDLNGATEYYQILVDIDEELKTLGEEADYVTQGRCSLFLMIMQVVVSFLHATSTYGTLCFIDEHVPLPKVFTLTFIDTCSLLLTATYCNYVSMVTRRYRRVNQILSEIIELESLH

>PxylGR3

SEMLIVGRLLIGINSGLNAGLAPMYLSEISPVSLRGSIGTVYQLVITITIVLSQVLGLSSVLGTSTRWSLLLALPLLPAALQCAALPFCPDSPHYLLFNKGNDKGATKALSWLRGGAEVNSEMEEMHQEAEKTKIRKKVTVRELFRNRQLRVPLITAAVVMLAQQLSG

>PxylGR4

YGIMMQKDFNVLDGIDCGVWIVWQILKIYVLGRAGSLLHIESRRIGETLHDLPVDKLESRTLLEIQHFSTQIRFKKMILTVYGYFPVDSTLLFNMVTAAMMYLLILVQFDFPEN

>PxylGR5

NKSSLEHNMYFAFSFTFLFVKALLVSFLGARIHSNSLVPLSMLFEVSSSNYDIEVQRFIDQVKHSHVAISGLDFFHVTRQMILTLVATIVTYELVLLQLNIDSIITADG

>PxylGR6

FGSLFWLGSFGFSAIHACVNDHTILRAYYDTKLKNYGDAYERITSLLYSETVILKVALQVSSCLPFTQYIVDIDKTLASRGVKVDYQH

>PxylGR7

YRMFDIGCSLGTITNCLFYASGCFSTIFMIRIGQRWPDTVQVIEAIERRLPPLPSRVTRQ

CNGIMIFVLTAALVEHILCDVYLYKEASACHDGDIWRKYFTDNIPWIYNYIPYSPWKAVV

TEMFNIHSTFMWSFNDALVMVCSIYLTHHFLNHNRLLENVMGQETFHLKEFRIQYKNMMN

LVNIINKEIGVVIVISFFCNLYWICMQLFNSLNKSDNSDNDFPSVECQQRSIANKSSLEH

>PxylIR1

EKDDNEYYLEEIYSFGTIKGGEINVTTIGHWNEKNGLQMWYSGYYKYLHRWDFNNLPLVM

ITVTSRPDRFRPEMLDYDNSSPDINVVTKTSVNIFNIVAERHNFRFVHSITDRWIGSLVK

NDTGTRAGVTSLFFREADFITVLRLYPELYSQIDYLYPHVTYIGTKFYYRIPETGVGKYE

NRFLTPLSPGSWSCILAMCVVCAGVLLTTTVVENRPSSAQFALFTVMALVSQQSFDDNDE

TVIERRSFARKVAVLVTGTSCLLLYNYYTSSVVSWLLSAPPPSIHSIDELMQSPLEVIFE

DVGYTKSWLQIPGYYFNKRYTHMEDKLRARIKSSKKGPLIVDVNKGIEMIKSGGYAFHAQ

IQNANERISKTFDKMELCKLGSLLSMPYNPLYPAIPKKSPFKEFFVWSLHRLNERGFISY

VQRRLASRDISCDGSIPRALALGGAAPAFALLALGGMLSLNIMVVEMVLFRISKRNKMTP

YLN

>PxylIR4

MKLTSFIYFLLCFYAFESVAAINMLERSRFINEFLDRHGQPALMISALNSPIYETLQLCK

ELSKGAKSFKARACFEGGDNRFNSECPRPDTIDKQNKKYDAKLPITDNFVYLGDLNNKRT

VELLLYSDRFYRLSNPARWILFYDSSGSKNDKNIAIYNASSNSNLDNEMEDIAFMLDEVN

LAINTEVYIVIEKQKELIVYLVHRRWMNLPLIWEDYGTWKPTTGFDRPHPETVLALRRRD

LQGLRVNCTSVILDNRSLGPMVMEDYMDRDVDSMSKMLYYLVVHLVEWVNGTRYLSTTDS

WGYLKPDGTFDGMVQEFWEGTADIAGTVLFPMKGRLPAVNYISPP

>PxylIR7d.2

MAILLIIFLWDCVTAIINPHGPTVVNDYVRCVDSIIDVNFHKAGLMYFMHTQNMSTSVARIRADLLREIHREREQIMVIIASPTEDDQPLCSSHREPRTDGRLLTADRFAPIPIANYVVVIVDSYTDFDKVVGRLVRARGWNPSALFLILYFHFASSNKINIEHAEDMVACLFSWNVFNIIVIVPETGNLRNALVYTWKPYDPPKYCGYSNETSRGRLKVVNTCKRGVVSAPNKILYHKLPRDMKGCFLTLLALERQPFVTMEITDPNVEKGFINELLKQFNFRPNYTFVNKPRGEETLGQWEGAISDLIAKKGQILLGGIFPDDEVHKDFECSSTYLADAYTWVVPRAFNIPPWAAPMIIFSDLVWYSTVAGFIVCVISWRTFGYLSGDSLYHNSIGHCFMNTLVSNLGFAAYARPVKFSLRMFFVVFSLYCILFVTGYQTKLIDVLTNPPFLYQIQTVEEMVESGLAFGGFEELHDLFQNSSDAFDCMIGAQWTDVADLHGA

>PxylIR7d.3

MYVSDLAIVTRGGDGNVKVTTYFPNNSQRCHDYRPVTTNASDVSEFFPEKFRNFYGCPLRVSLIEHAPFNIFTNSNGIFKIDGIEGNFVTLLCDILNSTLVPVFPAEKSFGVLKDGVWTGIMADLVYNRADISASSAIFTVERFKAVQVTHAYKHLDLVWCLPRVSQRRAWARVLLPLLSCTTALVCLTAAVFLSVAAVFNKIAIRNCEEQAPRLSVLQTCAVFLGQPVRWARAGGVLDQLFALWLWFCLVLRIVYQGELVRSLERGAPPPRVPTLARALQLVDQYGGADSVATFYRNTTILKKFKVLRLEEVHPMLGDIGKGRRFLLAV

>PxylIR8a

MEIAFLFLLVFLINLGCIASELSLRFVFITEVQEPELAQQIARALKVSENIRPELRLTDF

IVYLDRENEEESYRKLCSAVSSDVSMIIDLSWSPWESATQISEQTSIPLVRSLLGNQQLV

SALDDYLEVRNATDAALLLPSEIDVDKTLYHLLGSSNVRVWVHSGLTKDAARALKSMRPE

PGFHAIIGDNTFVTDTYRRAVKEKLVRRDYRWNLVLTDYSAANLDISMTVPTMILSADPT

ECCKLLGQAEDCSCPADIQRKQHILNALIAYISEVYFKLDTSIPTVSVSADCENLRMSAD

MNVTRDRLYRQFAEDAALSNDTIFYWDEDQMGLFLRTRFTLSSYAPSEGLNHVASWSADE

EFKLLPGVTLQPLRMFFRIGTTAAVPWTLPKLDPDTGQPMFTEEGQPMYEGYCIDLIQKI

SEVMEFDYEIVTPQTGTFGRKLPNGSWDGVVGDLMRGETEFAVSALTMTAEREEVIDFVA

PYFEQTGILIVIRKPTRKTSLFKFMTVLRTEVWLSIVAALILTGFMIWLLDKYSPYSARN

NPEAYPYPCREFTLKESFWFALTSFTPQGGGEAPKALSGRTLVAAYWLFVVLMLATFTAN

LAAFLTVERMQTPVSSLEQLARQSRINYTVVEGSTIHEYFINMKFAEDTLYRVWKEITLN

ATSDQAQYRVWDYPIREQYGHILLAINASGPVPDAKTGFEQVNEHTDADFAFIHDSAEIK

YEVTRNCNLTEVGEVFAEQPYAIAVQQGSRLQEDLSRALLDLQKERFLEQLASKYWNETA

KQACPDADESEGITLESLGGVFIATLFGLGLAMLTLAWEVFYYKRKEKNKIKSIDATVEK

PKEAFAPSKKSLLETKMAEGVARLRKRDKKGKGNLPKNVTIGDTFRPAADNANVSYIKVY

PKDGFKP

>PxylIR21a

VKELNEMKVTFDIIVISIALLFQVYVCEVVEYYPSLSKLSKPLQEKQKERQSIKYVPNKT

ANGHASDKKPKNKPINKREVDTAFRGHPKTREELWNEHFLNKSSAFDQNPSLIVLLHNLT

LRYLNDCTPVILYDSEVASEEEHLFKDLFKDFPVTFVHGKIDTDDKLINPDLLKPVRECV

HFIVFLTDLRTTSKVIGMQASSKVVIVARTSQWAVQEYLVSSLSRKFVNLLVIGQSFKDE

DDKLEAPYILYTHELYIDGLGASRPVVLTSWSHGKYSRDVELFPHKMQQGYAGHRFIIAA

ADQPPFVVRRVKSDLDGGNPRVIWDGIEIRILKLLGERNNFSIEIKEPQELHLGSSDAVA

KEIAMGRADVGIAGMYLTNQRIQEMDMTAAHSQDCAVFVTLLSTALPRYRAILGPFHWHV

WVALTFTYLIGIFPLAFSDKHTLRHLLNDSGEIENMFWYVFGTFTNCFTFVGKNSWSKTT

KITTRLLIGWYWIFTIIITSCYTGSIIAFVTLPVFPETIDSIDQLLRGFFRIGTLDHGGW

ERWFFNSSDPKTNKLLSRMSFVPNVEAGIRNTTKAFFWNYAFLGSKAELEYIVQANFSLT

KSKRATLHISNECFVPFGVSIGFPNQSLYTAKLSGDIQRMSQSGLLNKIVDEVRWEIQRS

TTGKLLAASSSGSIKIVSAEEKGLTLEDTQGMFLLLAAGAIIAAAALISEWMGGCNRKCR

PKKKINSADRKILSVNSRENLIPTPKSDVSSEIKFITDDDDVDSRVYFNQRPYSAGSRDT

LDGHTIHVTDETIIVHQSVDTDRWEYRRSSSMDLDKEVQEIFERDQRKRRFQSENILATN

NITRHHTASKSAFGDPVI

>PxylIR25a

MTASLLVILVVGGSLRLCTSQTTQNINVLLVNEESNALAEKAFEVAKEYVRRNPSLGLAV

DPVIVVGNRTDAKAFLENVCRKYNDMILAKKSPHVVLDFTMTGIGSETVKSFTAALSLPT

ISGSFGQIGDLRQWRTLTANQTKFMLQVMPPADILPESIRAIVTKQDITNAAIIFDEYFV

MDHKYKSLLQNIPTRHVITPVKSFNRDEIKNQLKSLRQLDIVNFFVIGSLRTIKNVLDAA

DENQYFGRKTAWFALSLDKGDISCGCKDATIVYLRPTPDAKSRDRLGKIKTTYSMNGEPE

VTSAFYFDLSLRTFLAIKSLLDSGKWPNDMKYITCDDYDGKNTPNRSLDLKAAFQEVKES

PTYAPFFIPEDEPMNGRSYMEFNTEISAVTVKDGASIGSRSLGSWKAGLANPLSLSDPEN

MSDYSAQLVYRIVTVEQQPFIIRDDDAPKGFKGYCIDLIEEIRQIVKFDYEITLAPDNNF

GTMDENGNWNGIIKELIEKRADIGLTSLSVMAERENVVDFTVPYYDLVGITILMKLPRTP

TSLFKFLTVLENDVWLSILAAYFFTSFLMWVFDKWSPYSFQNNREKYKDDEEKREFTLKE

CLWFCMTSLTPQGGGEAPKNLSGRLLAATWWLFGFIIIASYTANLAAFLTVSRLDTPIES

LDDLSKQYKIQYAPLNGSAAMTYFERMAHIEVRFYEIWKEMSLNDSLSDVERAKLAVWDY

PVSDKYSKMWQAMKEAGLPNSIEEAVQRVRDSKSSSEGFAWLGDATDVRYHVLTSCELQM

VGDEFSRKPYAIAVQQGSPLRDQFNNAILQLLNKRKLEKLKENWWNNNPAAMKCDKQDDQ

SDGISIQNIGGVFIVIFMGIGLACITLGVEYWWYKWRKRPTIGDVTQVTQVEPAKVTRNN

VAPADHHNTSKVGEGFSFRSRNLGLANLRSKF

>PxylIR41a

PIEILLQIIFHQYLNGSYCLTIVSENKLNAHMLNGTITYIDVNETQNLVDKILAVSEMGC

SDFIVQMSDPQKFVSAIEQVNHLGNARRADKIMIFIPMDKDSDPKVLVDLLLLKEMAFVA

NLILILPANSTKSTDQCESYDIITHKFVGADAQANDPLYLDSWDACTQQLKTNINLFPHD

ISNMQGKTLKLATFTYKPYVVLDLDNTTVPGGYDGLEMRVMAEFCRWVNCTLQIIRDDEN

EWGDIFEDGTGNGILGNVVEDRADFGIGALYSWHDSYVHLDFSASLVRSGITCVAPAPRI

IASWELPLMAFSWPLWGTVAGTFVYAAAAL

>PxylIR68a

IPYISECLTFLTPEALTDNSWKTLILPFKLYMWIAVLLVLLITGAIFYGLAKYYESLLKM

KDDDVIKSRSGKELEVYKNNSDLYEVQPAGLYLFGEIVNSILYTYGMLLVVSLPKLPAGN

SIRLLTGWYWLYCILLVVSYRASMTAILANPAPRVTIDTIKELVDSKVTCGGWGAETKKF

FVDSSDEMTHKIGERFESINDPYLAAKKVAQGVYAYYENIYFLKYLSVKRKNIALFNKFT

DEDTNSTSNGTAVEAVESPEMERNLHIMSDCVVNIPVSIGFQKNSPLKP

>PxylIR75d

MEFISFLLSYFASKNILFLTAFLCWSKEDILLLQRTASLRGLRVAVVTGPGLPRLEGDGQ

ERVQGYALDVDCPGAERVIAQASNTRAFNQRHSWFLFEDSAYNDTKVDSILSDAAILLDA

DVVWFSNDVAVDLYRVNIHEPLLTMRLNVPRSPPALQQYWRRLPSAVARRKDFGGVYVKA

VLVISHPESFRGWDDLSTKHIDTLPKATYPLVMLLADDMNFRYDFMQVQTFGGLVNGSFL

NSAVGVVEQGRAEVAATSMIFRRDRMRQTHFVTETYDLGPAYIFRQPPQSAVANIFLLPL

SRGVWVASGVVFSLIALFLAVFSKRLVKIDSSLAVITPGETFTFALGAVCQQGFHMTPHL

QSIRVMMFFSFITSLFLFTSYSAKIVALLQSPSTSIQTITDLAKSPLTCGVQDTPYKKTY

YLENPHPATKLLYERKLKPQGDRAFSYSVVEGVARLRTGQFAFQVEVSSGYDIISKTFTE

SEKCGLNYIDPFRLQTLGIPIKKHSGLKEIFSTRLRWYRDTGLMGRSARTWFATKPRCDS

GAAGGFVSVGITDCLPAIQVLGIGAMLAAALLLVEISVHKLYMKYSMKNQQDI

>PxylIR75p

LGCALLLYVTSKWEASMGMHPLQLDGSWADVLILIIGAVLQQGCTLEPRHAAGRVVTLLL

FISLTILYAAYSANIVVLLRAPSSSVRTLTDLLNSPLSVGASDFEYNRNFFKKLNEPIRK

EIYDKKIAPKGKKPNFYSMKEGIQKIREGLFAFHMELNPGYRLIQDSYQEDEKCDLKEID

YINEIDPWVPGQKRGHFRDLFKINFMKIRESGIQACIHHRLHVPKPKCSYGVSSFSSVGV

TDIYPALLITLYGFLVAPVTLLLEITYKQLLRVREKRKALEKIDFTE

>PxylIR75q2

MRRYWVVYFLCLLVGCRAEPVEEAAMVTDVIHAMERPSSVIATLCWSLKKKVRLYSSLAG

ENVTHINMMQFLRPDLTPERHDRDQQVVFLVDMTCPNITQYLEMSAKEQHFRSPFRWLLL

TKSEAADGSDDESIIPAAIRNIDVLLDSEVVLAIDLGNHSYVAHLIYRISAGSEWRTEHY

GSWDPKNRFQKGHLIDSAARRRANLEGYVISVCYVLTDADSVNHLMDGIDDHIDTITKVN

FPTTNHLLDFLHAGRRLVFTDTWGYRVNGSWNGMTGYLVRREVEIGGSPMFFTSERASLV

DYVSSPTPTRSKFVFQQPKLSYENNLFLLSFRTTVWYSSIALIFLLYLALLAVAFWEWKK

NAHQYKQREENASILRAKVSDVVLLVFGATCQQGSYVELKGSLGRVVMLV

>PxylIR75q2.2

IVIAAVCWDSKFKMNLTKSLGNLDKSILIKFYDEKTLFTRLLPEHHVVFLVDASCNSTKY

ILEKANNNTNFRRPFHWFILNKDGGETIPTEIEALDLLPDAEVAIVNRIRNESYVLYLVY

KISSKSVWRTEYYGEWSMYTGFHKATTLGTSTAMRRRDLERYVMPIAYVITNPDSINHFV

DRGDGEIDTLSKVNYHTTNNILYFMNARRRFKITNTWGYEVNGTWTGLTGFMVRKEIEFG

GTPLFLTAERISVIDYLACPTLTLSKFVFKQPNLSYGNNLFLLSFQTAVWYSSLLLVCVF

FLVLFVVAFCEWNEKGADYLERQKDASILRAKMADVVILVLGAACQQGSSVELRGFSGRF

VIFVLFLALMFLYTSYSANIVALLQSSSSQIQTLDDLLNSRMKCGCHDTVYNRHYFSTAT

EPVRKAIYQKKVAPAGSKPQFLSMEEGVKNMQKGLYAFHMETGVGYKFVAKFFLESEKCG

LKEIQYLQAFDPWSASRKHSPYREIFKLALLRIREHGLQSRENLLLYEKQPKCTSQNGNF

VSVSMVDCYPVLLILCYGVFSAILLLAIEILYHRRTEMIKRLGFRGFWVSE

>PxylIR76b

MAGIELLISSICYNATFCDAYYDNSGLDERLTKKQNEFLALRSEVNGKHLRIATYNNYPL

SYVEEINGTLVGQGVAFVIVDILRKRFNFTFDVILAEKNYESGGSKPEDSVIGLVNSSIA

DFAAAFLPILYDYQTKVSFSHLLDEGVWLMMLKRPKESAAGSGLLAPFEYEVWYLILAAV

LSYGPCITLLTKLRNKLVTDEEPPIPISPSFWFVYGAFIKQGTTMSPEANTTRVLFATWW

MFIILLSAFYTANLTAFLTLSKFTLDIEYPQDLLRKNYRWMAQEGGTVQYIVRDPNEELH

SLNVMVKNGRAEFRAISNDFDYLPLVQGGAVLVREETAVLHLMYGDYLRKAREGVEEADR

CTYVVAPNAFMTTMRAFAYPRNSTLRRLFDPVLNYVEQAGLIKHHLHRDLPSTKICPLDL

QSKDRRLRNSDLLMTYLIMLAGLAAATAAFMGEMIFKRYIRVKWKVKVHSGLIGQKKKSQ

SKKKETKKRWTDDTKPPPYESLFGRNSRYTGSENTEHKVINGREYWVVNTISGDRRLIPI

RTPSAFLYERK

>PxylIR87a

RWHSGSPAMFTKEILLILLSLHFISRVQSENPLLTTDGNSDQIAEAAECVLKLSAKYFVE

KKALSGSIVIININSYVSTTQGLLLQTIHGGTKYSVMVKDSFYKHANASHFPEKAKNYML

ILEEKSELTRNIKQLNKLPTWNPLAKAVVYYELEGNETGPEVAINFINELRSHKLFKSII

FIYSPANEEVVAYTWRPYGGDNCGGECTSVYVLDRCSDGDLQQVEKQSEMFPADMRGCPL

VAYAVVSEPYVMPPVMRLTNTSYPDAYYFDRGGEINLIKIISEFTNTTLIIRTSDIPENW

GVIQPNGTATGAFEILRQDEVDLVIGNIEVTRTIRKWFDPTVSYTQDEMTWCVPRAGQAP

TWDNLVIIFQWSTWVATFASLIIMGLVFHYLYYKQNNGNVTKWPTNSLLMTFSMLLGWGA

NFEPRSATFRILIFGWLFFSINMGISYESFLRSFLMHPRYMKQISTEAEIIESRIPLGGR

EIYRPYFETNNASSFYLYRKYNSTTFRAGVERAARGGFAVVSSRRQAEYTDQRLGRGAPL

IYCFPESHNLYKYGVVLLARKWFPFLERMNGIIRSVSENGLIDKWNSELFIHAAGADGAA

ASVALGVGHLLGAFWFIGFMYAAAVLLFAAELA

>PxylIR93a

MQVWTAVVVSLCVTWRLVGAEDFPSLITANGSIAVVLDRQFLGEQYQLVLDQVKDWVREL

ARVELKHGGVVVHYYSWTTISLKKGFIAVFSIASCQDTWTLFSRTEEEELLLFALTEADC

PRLPSDAAITVSYMEPGHELPQILLDLRTTKAFHWKSAVILHDDTLNRDMVSRVVQSLTS

QIDDEDVPAISVTVFKMKHEINEYLRRKEMHRVLSKLPTRYIGNNFVAIVTSDVMSTMAE

TVRDLGMSHTQAQWLYIVSDTNTHTGNLTSLINALHEGENLAYMYNITDDHPDCKNGLIC

YCQEVLKAFVSALDAAVQEELEVAGQVSDEEWEAIKPSKLQRRNMLLKHMQQHISVNSRC

GNCSSWRALAADTWGATYRAFTEDTAAPGRKKDADNDTTSGVIEQIELLQVGTWRPVDGV

RYHDALFPHVEHGFRGKPLPIITYHNPPWTFLHTNESGTIVSYSGLIFDIIDQLSKIKNF

TAKILLPGNVKHDFSNDTVDSMQSESALATLSAVARGQAALAAAAFTILSDPMPGINYTV

PVSTQPYCFMIARPRELSRALLFMLPFTTDTWLCLGFAVILMGPTLYIIHRLSPYYEAMG

VTPQGGLSTIHNCLWYIYGALLQQGGMYLPRADSGRLVVGTWWLVVLVVVTTYSGNLVAF

LTFPKQEQPVTTVAELVGQRGTFTWSIRKGSYLESELKNSDEQKYVTLLKGAELLTSSPE

IGLAGSGARVLHRVRTQRHALVDWKLRLSYLMREDHLRTDTCDFSLSAEEFMAEQVALIV

PSGSPYLSVINKEIMRMHKAGLISKWLSAYLPKKDRCWKSSAVAQEADNHTVNLSDMQGS

FFVLFLGFFIASSVLLLEWLYKRHKKKSEEIVIKPYVE

>PxylGOBP1

MERRWCLLVLAAAAAAGLPGVVRGTVEVMKDVTLGFGEALEHCREQSQLTEEMMEEFYHFWREDFKFEARAVGCAIHCMSRYFNLLGEQQRMHHDNTHKFIQSFPNGEVLSHQMVGIIHTCEQQHDAEQDDCWRILRVAECFKRESQAQGLAPSMEMLMAEFIMEADV

>PxylGOBP1.2

MERRLRLVRVLVAASLPMLVLSSVEVMKDVTLGFGEALKHCREQSQLTEEMMEEFFHFWRDDFKFEQREVGCAIHCMSHYFNLLDDTHRMHHQNTHKFIKSFPNGEVLSQQMVGIIHTCEQAHDKEPDNCWRILRIAECFKKECQAQGIAPTMELLMAEFIMETDV

>PxylGOBP2

MASVWSLVVCGLMMAALPAARGTAEVMSHVTAHFGKTLEECREESGLSGEIMEEFHHFWREDFEVVHRELGCAIICMSNKFQLMQDDARMHHENMHDYIKSFPKGDLLSETMVRLIHNCEKKYDDIDDECSRVVKTAACFKKDAQAEGIAPELTMIEAVLEKY

>PxylPBP1

MITKKLACLMMVLMCALKKVESSADVMKGLSENFGKALGDCKKELDLPDSIMTEFYNFWKDDYVLSDRSTGCAIICLSSKLDLLDPDGNLHHGNAKDFALKHGADEGMAGQLVGMIHECEKAAPDNPDACLKVLDIANCFKKKIHELKWAPSMDVVVAEVLADV

>PxylPBP2

MADSTVSRWRRFALCALLVAIQVKKSESSADIMQKLTIGFTEALEHCKKDLQISNEVMQDFYNFWREDYALVNREMGCVLMCMAARLDLVTEDLKMHHGNAHEFAKKHGADDTMAKQLVTIIHECEQGAASVPDECARTLEMAKCFKTRIHELKWAPSMEVVLEEVITELKN

>PxylPBP3

MWFPVKLGAVVLLTLAAMDAEGSKETMKDITSGFFKVLNECKHELNLPDHLVGDFYHYWRQEYALLDRDLGCAILCMSRKLELIDASGKLHHGNTQEFAEKHGADNSMASKLVEVLHSCEKQHEAVSDDCQRALEVAKCFRSSVHELGWAPTIDVIIEEVLTDM

>PxylOBP2

MLFAVVLVSAFATLCVGQQVPNNTGPAPVLCGIIPKTIKGCLDFPSIVEQKLSKGCGNTQTVCESLKCVFEKSGWIKKHQLVKSKVISDLDKFAEEHPAWYNATQAVKATCVAQDLPAQGVFINCPAYDVLHCAQILYYRSALPEQWSTSPDCLYPRQFTAACAFCPEDCFSPQIPYRSCNACLNYPNRA

>PxylOBP3

MFLASLATLVLITFTLSSGKMKAEEPRTREMPDMSSSSSVSFHDGNDEHAEDDMMGIMTHCNETFRFDPAYWQSLNESGTFPDENDKNPKCFIRCVLEGTRVASLDSVFDAARAAEVFAGERGGRPMDDLETLAKNCADDRRETCKCERAYGFMKCLMEAEIETYEVYKADDS

>PxylOBP4

MKMGSSMYVAFLAVAVAALFGNTHAISDENREKLKKEMGPIFMECAKEGSLNLDDLKQYKGVKELPADEGVTCFFACAFKKIGMIDDKGMFAVEESVERGKKYMDSEEKQKHLEEAANTCASVNDESVSDGDKGCERAKHLYECLIKQAEKFGLELPTSAV

>PxylOBP5

MGSPVLVVFLTGTVLAILHSTNALSDDNKEKIKQDMVPIVMECAKEQEINPEDLKELKGAKTLPEDKDMMMPCFFACTFQKNGMMDDNGMFLPEATIKNGKKYAENEEEEKKMEEIAKACAPVNDESVSGDKQKCQRASLLYACLAEQAEKHNIKMHE

>PxylOBP6

MIRGVVLCCAIYFLSLSLPLVKSMTDEQKAMIHEHFEKVGLECLKGNMITEDDIKNLRARKVPEGENAPCFLACMFRSIGIIDDKGLMQKENALELAKTVFKDPEELKMIADYIHSCSHINSEAVSDGEKGCDRSINAYKCMVENGKQFGFDV

>PxylOBP7

MFVESPCILISVLVFILISVAKCENHNESPITSVVRKGLVATARGCSAQVGAADGDLEHLRGDPPFPEKAACVVKCLLNKIGIVKNDKYSKSGFLTIITPLVMANKKKLEHMKSVADNCDQEVNHKEATPCQLGNEITACIFKYAPELHFNK

>PxylOBP8

MIKTCLFISVCAMVNVLLVQARTEKEIREEFIQLGMECAKQHQVTPEEIQLMHQHVIPDGRGARCLVACVFKKKDLINDKGMLDIDAAHSMADKEHLDDPTMIENSKKLFDLCKSVNDETVSDGEKGCDRAALLSKCLIANYSKFGFKI

>PxylOBP9

MFPYIFTYVFIGVYFTCTTGTLMKDEERVKFHKTIQSCAQQHPISPEDQGSLVNWQISDTQEVKCFIACIFQGIGMIDEKGRFDAAHVNDITKLMMTEDDPDVLQQTQDITESCKYVNDRHVGDPHETCERAASLFRCAAKWTGKKDS

>PxylOBP10

MHYSSLFVIVLVASIIGLCASVKQKKVNLAPDKTARLVQHAIDCVAATGVSPEVLMQFRQGHVGSDDKSQQFVHCLLKRLSLVSKDGHGKTQRVLDLYPDNVDKEGIKKAFDECNKLNGSTPTETSFKVFVCFHKTSPVVIGL

>PxylOBP11

MFVKNHWLIVMVVATGICVVSSITRKQMKNTSKIMKKSCMPKNNVTEDQVGNIEQGEFLEERKVMCYIKCIYAMGGAIKNDKFVYDAMIKHVNLVFPPEIKEPTLAAINQCRDVDKQYADSCEAAYWVAKCMYEYGPEHFFFP

>PxylOBP12

MERWEFGLLIIAVILTSGAEAMTRAQLKNSAKMMKKSCLGKVDVPEENIVDIEKGKFIEEKNVMCYIACIYQMSQIVKNNKLSYEASIKQVDLMYPPDIKDAVKKSIEQCKDVSKKYKDLCEASYWTAKCIYDHDPKIFIFA

>PxylOBP13

MSTRRLLLFSMLIAAVFGGKSKPEFSEEIKEIIQHVHNECVGKTGVAEDDITNCENGVFKDDQKLKCYMFCLLEEASVADENGVVDYEMMISLIPEDYTERVSKMIMACKHLDTPDKDKCQRAFDVHKCSYEKDPDLYFLF

>PxylOBP14

MVLLVLIAKYVLFVALCDAMTMKQLKNTGKMMRKSCQPKVNAEDAQIDGLKDGVFLEEKETMCYIACIMKMANAIKNGKLNYEAAMKQADLLLPEEIKEPAKAAITSCRKVADQYKDICEACFYSTKCIYTQNPDIFFFP

>PxylOBP15

FIVLFVVTFVCYSEAMTDEEAKLQFTKIAMNCTKKYPLEVHEILDLQKLKVPTKKTAKCLLACAYRLEGSMNEKGLLDYEHMMKTADLLADGDEKRLKNAKAVADICIKVNDETVNDGEAGCDRAALLFKCATETAPKYG

>PxylOBP16

MERLALLLAVLAAVRAEFPTKEFVEMLKPVILKCEEKTGVNKDFVDQFNKGTMVDDPTFKCYLKCMFLEFEVLDPTSGHFRYEKMLGILPQEMKPIAMEMGKNCIHFKGEEGSDLCEVSYQLHQCWQKASPQHYFLLRR

>PxylOBP17

NLNFFYFKMTRPELKKILSEAKRQCMASNGVTEDEVGNIEQGQFLEERNVMCYIKCIYVSGGVIKKDIILHDAMIKHVEKLWPQETKASIIDAINHCRYVDEKYADACEDAYWMARCIQAYTPEHFLFP

>PxylOBP18

EEQVGDIEKGHFIEERNVMCYVACIYSMSQLVKNNKLNYESVIKQVDMMFPPELREPTKAAAEKCKDVVKKYKDLCEHSFWTAKCMYDVDPKNFVFP

>PxylOBP19

MYNSCVLFLLLCVSLTHGNLFLEELRKKGASLKPLSACCDIPDLGDPEHLAACSSPKLQGPCND

>PxylCSP1

MKAAAFIALFLIGKAVCEDKPTYTTKYDNIDLDEILSSERLLTGYVNCLLDQGPCTPDGKELKHTLPDAIDNDCRKCTQKQKEGSDRVMGYIIEYRPNDWAKLEKKYLSDGSYKKKYLEKKNASENNGDSKSTEAKNKDDEEKKGKGDGEEK

>PxylCSP2

MQKLTLACLLVAVAAAAARPNDSHYTDRYDNVNLDELISNRRLLVPYVKCVLDQGKCSPDGKELKEHIQEALENNCGKCTDKQREGTRKMIGHLINHEQEFWDQLIAKYDPERKYVSKYEKELKEVKA

>PxylCSP3

MNSLVLVCLALAAVAAARPQATYTSKYDGVNVDEILANDRLMMPYIKCALDHGRCSPEAKELKSHIKEALENNCAKCTDKQKPAVRKVIAHLINHKPAEWRQLSDKYDPAGKYTAQYEDQLR

>PxylCSP4

MQTVTLLCLLAAVAAAAAAPADTYDAKYDSFNAHELVQNQRLLKSYGKCFLSKGPCTAEGSDFKRVIPEALKTTCGKCTRKQRELVRVVVKGFQEQLPQVWTEIVSKEDPKGEYKDSFAKFLEGSD

>PxylCSP5

MTSLKLLALACVMAAALADDTYTDKYDNINLQEILDNKRLLMNYVNCVLEKGKCNAEGKELKDHLEEALQTGCAKCTEAQKKGAQTVIEHLIKNELELWRELANKYDPQGTFRKKYEDQAKEHGIVIPDE

>PxylCSP6

MRVLMLVALSCAVGAAWAKPASTYTDKFDNVNLDEILESNRLLKGYVDCLLDKGRCTPDAKTLKETLPDALENDCSKCTEKQKSGSDKVIRHLINKRPELWKELAVRYDPNNIYQDKFKDKIEAVKTVKA

>PxylCSP7

MTIWALLAVVALASAALAQQQEYPTNTKYENFNTDSIIANERILLGYYKCVMDKGPCTKDGKNFKRVLPLTITTACSHCSPKQKTVVRRLLLGIRAKSEARFMEMLDKYDPGRLNRPALFTFLANGN

>PxylCSP8

MKLFVALCFVTLVAYSSARPNGSYTDRYDNLDLDEILNNSRLRVPYVKCLLGKGKCSPDGKELKSHVREALENQCGKCTPAQQAGTRKVIGYLINNEAGYWQELVALYDPQRKYVKQYETELRKVSG

>PxylCSP9

MKLLIITLALAATCVAAQKRYTNKYDNMDLDEVLANRRLLVSYIKCVLDQGRCTAEGKELKTHISDALQTGCKKCTPNQREGARRVISHMIKEEPEYWTMLVEKYDPERMYSTKYEKEINSIQ

>PxylCSP10

MNSLLLFVCLSLAGLAASEQYTDRYDNLNVEEILSIRRLLVPYIKCMLEQGRCTPEGKELKLHVKDGMQTGCSKCTPWQRTNARKVVKHIREKETEYWEALKKKYDPKDEFKPTYEAFLAADD

>PxylCSP11

MRSLIILCCLAAGALAADKYNPKYDNFDVETLISNDRLLKSYINCFLEKGRCTPEGSDFRKALPEAVETICAKCTEKQKTNIKKVIKAIQQRHPKQWEELVKKNDPTGNHRDQFEKFIHDKS

>PxylCSP12

MKVAIIVSMLVVAACAQDKYKSDLEGDFDVTELLNNERLLLSYTRCLIDKGPCTPEVKAVKDKLPEALATKCAKCTDKQKELGKKLAVELKRTHPAVWAQLVAKYDPQGQHQAAFQEFLTKQ

>PxylCSP13

MKLLLLSLSLLALSQGAPYSAEHDDFDVEPVLRDPALLKSFSDCFLDKGPCDDIQSHFKKNIPDAVQTACSSCTDKQKHLMRRVLEVLTVQYPDVAEQFTTKYDPEKKYIPALLAAVDKA

>PxylCSP14

MQFSITHLLVLSALVAVCWAQAKETKPRVSESALEEALNDKRYIQRQLKCALGEAPCDPTGKRLKTLAPLVLRGACPQCTPQETKQIQRTLSYVQRNFPQEWAKIVRQYAG

>PxylCSP15

MRRILAAALAAGLIALASTAVDLNNLEDMDVEALFRDKARSAAAFQCLMDQGPCGELQPLRDSLPSMVETQCANCSPKQREKYVQVNLLLLAQYPAEYEKLALKYSPKLD

>PxylSNMP1

MKLPKHLKFAAGAGGAFLFGILFGWVMFPAILKGQLKKEMALSKKTDVRKMWETIPFALNFKVYLYNYTNPEEVQKGGVPIIKEVGPYHFDEWKEKVEIEDHEEDDTITYKKRDTFYFNQEKSGPGLTGEEVITMPHVFMLAMATVVSREKPAMMNMIGKAINGIFDNPADVFIRVKALDIMFRGTMINCARTEFAPKAVCTALKKEAVNGLVMEPNNQFRFSLFGSRNGTIDPHVVTVKRGIKNVMDVGQVVAIDGKPQQDVWRDHCNEYQGTDGTVFPPFLTEHDRLQSFSGDLCRSFKPWYQKKSFYRGITTHRYIANIGDFANDPELNCFCDGPCPPKGLMDLMKCMKAPMYASMPHFLDSDPELLKNVKGLNPDVNEHGIEIDFEPISGTPMVANQRVQFNMQLLKHDKVELLNNLPDTIVPLFWIDEGLALNKTFVNMLKFQLFYPKKAVGVIKWLLVTFGGFGLIGCTIYHYKDRIMSFASSPGSAAVTKVKPEEVEQKDVSVIGQPQEPAKINM

>PxylSNMP2

MLGKHLRLGFAISLVVLVVAILMAAWGFPKIVQTQIKKNIQLADGSLMFDKWKKLPMPLTFKVYVFNVTNPDAVDSGERPRLQEVGPFVYKEYREKTILGYGENDTIKYTLKKTFIFDQEASGSLTEDDVLTVLNFSYMGALLSVFEIMPGLIPMINQALGVMFQDLTDPFLRVKARDLFFDGIYLNCVGESSALGIVCGKIRADAPPTMRPSEDGNGFYFSMFSHMNLSEAGPFEMVRGTEDVSQLGHIVSYKDKTSMNTWGDKYCGQLNGSDSSIFPPIDRSRVPERLYTFEPDICRSLYASLVGETTHFNMSAFYYEIHESALAAKSANRENKCFCRRNWSANHDGCLLMGLLNLMPCQGAPVIASLPHFYLASEELLEFFDGGVKPDKEKHNTYVYLDPVTGVILEGVRRLQFNLELRNMKGVPQLENVPTGLFPLLWIEEGAILPQSVIDELQHSHTLLSYVEAVRWIVLGIAILAFLGLAIAVIRTGNIPIFPKQANSVSFILRPGSFNPTDAHKAQ
