# Supplementary material for: Identification of Genes Involved in Chemoreception in Plutella xyllostella by Antennal Transcriptome Analysis
Source: Sci Rep. 2017 Sep 20;7:11941. doi: 10.1038/s41598-017-11646-7 (PMC5607341; doi:10.1038/s41598-017-11646-7)
Supplement: Supplementary file 2 — Supplementary Table S1 [file 41598_2017_11646_MOESM2_ESM.pdf]

Identification of Genes Involved in Chemoreception in *Plutella xyllostella* by Antennal Transcriptome Analysis

Shiyong Yang[1], Depan Cao[2], Guirong Wang[2], Yang Liu[2]*

Supplementary Table S1. Primers for RT-PCR expression analyses of *P. xylostella* ORs and IRs

Gene Name	Forward Primer (5'-3')	Reverse Primer (5'-3')
**ORs**		
PxylOR2	CTCACCGCCAACACCATCAC	CAGCAGCCACGAGCAGAACAT
PxylOR1	CTCCCACTTCTTCACCATGTACC	CATCCTCCCAACTATCACCATC
PxylOR3	GCTGAGATTTCTGCGTATTGGG	ACGCAGATGCTACACGCAGTTAT
PxylOR4	GCCCAGGACTTGCTCTTGTC	CAGGGTTTCGGGATCAGGTC
PxylOR5	TGTTATCACAAGCACAAGGGAA	ATTCATCGTCGTAGATATGTAGAAGTG
PxylOR6	ATGCAGATGACGCTGATGGTA	TCAATGGAGCAAACTGACACG
PxylOR7	TTGTGGCGTCACTCACTGTTC	TTGTAACTGTTGAATATCGGTATTCC
PxylOR8	GATCGGTCATGTTTACATTACGAC	TATTAGGAGCAAATCCAGAGTGC
PxylOR9	GCTTCAGTATTAGTCGTGGCA	AATATCTGTCGGAGAAAGGAGA
PxylOR10	GCACGCATTGCAGGAACAAGA	GGACACCACCGACAGCAGGAT
PxylOR11	ATAGGGATATGATGCGTCACAAGG	GTGCGGTAGTCAAACGGGAGT
PxylOR12	AAGAATCTGATCGCGTTCACC	CCAGGAATAGGCAGCAAAGTA
PxylOR13	GCCGTGTTGGTTTGGAACTC	ACTGCATGTGACGCAGAAGA
PxylOR14	TTTTAGTGTTTCTATACGTGCTGG	AGTCCTCACTGAGCGTGTTGT
PxylOR15	GGCGTCTCAACCCATATCCAC	CACCAGCAAATACATCAGTGTCCA
PxylOR16	TTTCTTCGGGCTGAATGTCTC	AGGCTCAAGCAGCACGATTAC
PxylOR17	GCACTTATTTATCCCAACCCG	GACCAACCCTTTCTTTATGTATG
PxylOR18	AATTACCGGCATTTGGTTTCG	TTCACCGATAAATATGTCACTGTCC
PxylOR19	CCAAAGCGGATATTGAAGCC	GCGTTGGTGTCATAGGAGGAG

PxylOR20	TCTACTTTCTTTACGATGGGCTTCT	ACCCAATACGAAGTACGCACG
PxylOR21	TACATTCTCGTTACTAATCGCAGAC	GGTAAACATTCCACCTTCCCT
PxylOR22	TCTGTGGCCGCTTATTTTACA	CAGGCCCATGCTACAGTGTTA
PxylOR23	CGGTTGGAAAGTGTTAGCCCTGTG	GTTTCGCCCGCAAATTCATCG
PxylOR24	CTGTCGTCTTGCGGTACATCC	GGGTAAACAATTAACTTCCCTTTC
PxylOR25	CTGATCCCGAGTTTCCATCCT	AAATGCCAGTTTCGTCAATCC
PxylOR26	CTTCTGGCTCCTAACCTGTGG	CGAAATTGTCGCTCAACTCTG
PxylOR27	TATCGGCACTTACAACATCCG	CATTAGCAGGCTCGTCATTCG
PxylOR28	GAAGCTCCTAAATACTATGAAAGG	TTAATCTCAACGGGAAACAAC
PxylOR29	CAACCTCATTGGTCCAGACAC	CTCCGAACAGGCAAATAAGAA
PxylOR30	GCTTATCGCAAGCTACGCG	GTGACCGTGAAAAATGAGTATAGAAAC
PxylOR31	ACCACGCAGATACGGATACTGA	AGCTCCACGACTATGCACGAC
PxylOR32	TTGCCTCTACTGCTGATGGTG	AATGAACCCGCTCAGACAAAT
PxylOR33	GTCCAGTATCGTCCTCAACGGC	TGAAAGCGGTCATGCAAACAA
PxylOR34	TCTCCCTATGGCTCAATGTAAC	CACCGAGGACTCACTCAACTC
PxylOR35	CAATTATTATTTCTTGAGCGACGAG	CAGTAGCAAGGAGGCGAGGAT
PxylOR36	CTTTGAGGATTCTGGCGTTTG	AGATATGGCGATCTTGGTGGA
PxylOR37	GGCAATAATCTGGTACAAGCAGGAC	CCGCTACAAACTTCAAACGCATAA
PxylOR38	TATACGTGAATGGGTGGGACG	CCTCCAAACAGTGTTGCTGCTA
PxylOR39	TAGACATCGAGGCCCTTTACC	GCACTTCAGTTTCAGGAGCAA
PxylOR40	ACTCTGCCATTTCATTCATGTTTCT	AGCTCGTAAGCTGGTGACTTCG
PxylOR41	CCCACTATTTCACTGCTACGACC	GCAAACCTTACTATTTCCCGATG
PxylOR42	CATACGAGTTTTCGGTGATTTG	ATGACCATGCAAAGAGCTACAC
PxylOR43	AGAAACGTCTTTGGACCACCT	CACTGCTCTTATCACCTGCTCAC
PxylOR44	TCGGAGTGCCCATCATGTTCT	CCCGTCGTAAGCCGTGTAGC
PxylOR45	GTCAAATTGAAGCCATGTTGATC	CGGCGACTGTGGTACTGTAGG
PxylOR46	AGGATACAGCCAGATGAAACG	TTGCTCACATACAGCACGATG
PxylOR47	GCAGGTGATCCTGTGGCTCTA	TGCTTTCTCCTTGCTCGTCTC
PxylOR48	AACGATGGAAGGGAGTGGAGA	AGGCGTGTTGATGAGATGTAGC
PxylOR49	TGTGCGGATGCTCCAAGTGAG	GTGGTTGGCGAGGATTTCCCT
PxylOR50	TACGTGCCGTTCGATAAATACAGT	GGCGAGCTGGCTCTTACAAAG
PxylOR51	CGGCTACTGGCTCTACGTGCTG	CCCGCCTTAGTGAGTTGAATGG

PxylOR52	CGCCTAATACAGAGTCTGGACGA	GTAGAATGCTCAGTTGAGTTTTGCT
PxylOR53	TTCGTTGGCATTTCCATCTTT	CAACAGCACCCTCACAAACCT
PxylOR54	GCGAAATCTTCAATTATTTGCC	TGGTTGTGTCTGGTCCCTGG
**IRs**		
PxylIR1	CAATATCGTCGCAGAAAGACACA	GACACCAAAGCCATAACAGTAAAC
PxylIR4	CCTGCCTTAATGATTTCTGCGTT	CTGTTTCTGGATGAGGTCTGTCG
PxylIR2d.2	AGAGAGATTCATAGAGAGCGGGA	TGGTAAAGTATTTTATTGGGTGC
PxylIR7d.3	ACCTTAGTACCAGTATTTCCCGC	TCCTCCCCTTGCCTATGTCC
PxylIR8a	TGGACAGAGAGAATGAGGAGGAG	GACACAGAAACCGTAGGGATCG
PxylIR21a	CTGTATATTGATGGTCTTGGGGC	TTGTGGTGATCTTCGTGGTCTTA
PxylIR25a	GTCGCTACTGGTGATTTTGGTG	TATGGACTCTGGGAGGATGTCTG
PxylIR41a	TTCATACCGATGGACAAAGACAG	AATTCACCCACCGACAAAACTC
PxylIR68a	TCTTGGAAAACGCTGATCTTGC	CTTTATCGTGTCGATGGTGACG
PxylIR75d	CCATTCCTGGTTCCTTTTTGAA	GGAGAAGACGACACCGCTG
PxylIR75p	GCACTGCTTCTGTATGTGACTTCG	ATTGGGTTTCTTCCCTTTTGGT
PxylIR75q2	GACCTGCCCTAACATCACGC	CCTCCAATCTCCACCTCCCT
PxylIR75q2.2	TGTGGATAGAGGTGACGGTGAA	CCGAGTACGAGGTGTAGAGGAA
PxylIR76b	TGCCTATTACGATAATTCGGGA	AGCAGGTGAGAGAAGGACACCT
PxylIR87a	ACGGAAATTCTGATCAAATAGCTG	GTGAGTCTCATGACGGGAGGC
PxylIR93a	CTGTTGTGGTCTCCCTCTGTGTG	CGTTGATCTCGTGTTTCATCTTG
**Reference genes**		
PxylRPS3	GGTGTCCTCCGCTTCATCAT	GTCACCAGGATGTGGTCTGG
